# Tumor-associated macrophage-derived exosomes transmitting miR-193a-5p promote the progression of renal cell carcinoma via TIMP2-dependent vasculogenic mimicry

**DOI:** 10.1038/s41419-022-04814-9

**Published:** 2022-04-20

**Authors:** Qing Liu, Enyang Zhao, Bo Geng, Shan Gao, Hongyang Yu, Xinyang He, Xuedong Li, Guanglu Dong, Bosen You

**Affiliations:** 1 Department of Radiation Oncology, The Second Affiliated Hospital of Harbin Medical University, 150001 Harbin, China; 2 Future Medical Laboratory, The Second Affiliated Hospital of Harbin Medical University, 150001 Harbin, China; 3 Department of Urology, The Second Affiliated Hospital of Harbin Medical University, 150001 Harbin, China; 4 Department of Pathology, The Second Affiliated Hospital of Harbin Medical University, 150001 Harbin, China

**Keywords:** Tumour angiogenesis, Cancer therapy, Non-coding RNAs

## Abstract

Previous studies have investigated whether tumor-associated macrophages (TAMs) play tumorigenic and immunosuppressive roles to encourage cancer development, but the role of TAMs in regulating vasculogenic mimicry (VM) in clear-cell renal cell carcinoma (ccRCC) cells has not been completely clarified. We conducted immunostaining of the tumor-associated macrophage biomarkers CD68/CD163 and double staining for PAS/CD31 in ccRCC human specimens to find that higher TAM infiltration was positively correlated with VM formation. Then we demonstrated that TAM-derived exosomes downregulate TIMP2 expression in RCC cells to promote VM and invasion by shuttling miR-193a-5p. Mechanistic analysis indicated that HIF-1α upregulation in macrophages could transcriptionally increase miR-193a-5p expression. Exosome-shuttled miR-193a-5p then targeted the 3′ untranslated region (UTR) of TIMP2 mRNA to suppress its translation. A preclinical study using an in vivo orthotopic xenograft model of ccRCC in mice substantiated that TAM-derived exosomes enhance VM and enable tumor progression, which confirmed our in vitro data. Suppressing TAM-derived exosomal miR-193a-5p successfully inhibited tumor progression and metastasis. Overall, miR-193a-5p from TAM-derived exosomes downregulates the TIMP2 gene to facilitate the development of RCC, which provides a novel perspective for developing therapeutic strategies for RCC.

## Introduction

Tumor-associated macrophages (TAMs) play critical roles in remodeling the tumor microenvironment (TME) to facilitate tumor development [1]. There are two general types of polarized macrophages, M1 and M2 macrophages. Unlike the M1 type, which plays proinflammatory and immunostimulatory roles, M2-polarized macrophages closely resemble TAMs, wielding protumorigenic functions [2]. Many published papers have revealed that TAM density is strongly associated with a poor prognosis in various human cancers [3, 4], including renal cell carcinoma (RCC) [5]. Therapeutics targeting TAMs have shown potential and could reveal relationship between tumors and TAMs [6, 7]. However, the specific mechanisms of TAMs fostering progression and metastasis of tumors and the communication between TAMs and tumor cells remain to be comprehensively investigated and clarified. In RCC, TAMs have been reported to promote migration and tumor growth [8, 9], and whether targeting TAM-mediated vasculogenic mimicry (VM) could be a prospective approach for the therapeutic intervention of RCC metastasis remains to be explored.

VM is demonstrated as a de novo pattern of tumor perfusion. In contrast to typical tumor angiogenesis, VM involves the formation of cancer cell-lined channels [10]. VM plays a key role by fostering a favorable environment for tumor cells and promoting metastasis, which is associated with a poor prognosis in many cancers [11–15]. Our previous study on VM in RCC also provides evidence to support its tumor-promoting role [16]. Moreover, VM replenishes the tumor vasculature and offers an alternative mechanism for innate or acquired resistance to antiangiogenic therapy [17]. Therefore, blocking VM might be a novel and supplementary antiangiogenic therapy for RCC.

In this study, we demonstrated that TAMs promote VM and cell invasion in RCC by transferring miR-193a-5p to RCC cells via exosomes, ultimately facilitating the metastasis of RCC. Further investigation of the mechanism revealed that miR-193a-5p, transcriptionally regulated by HIF1-α, directly targets the TIMP2 3′ untranslated region (UTR), thus downregulating its expression. Our findings revealed a new picture of the interaction between tumor cells and TAMs: specific miRNAs transferred from macrophage-derived exosomes (MDEs) to RCC cells downregulate TIMP2 expression and promote VM and cell invasion in RCC.

## Results

### TAMs are associated with VM in ccRCC patients

**Fig. 1:**
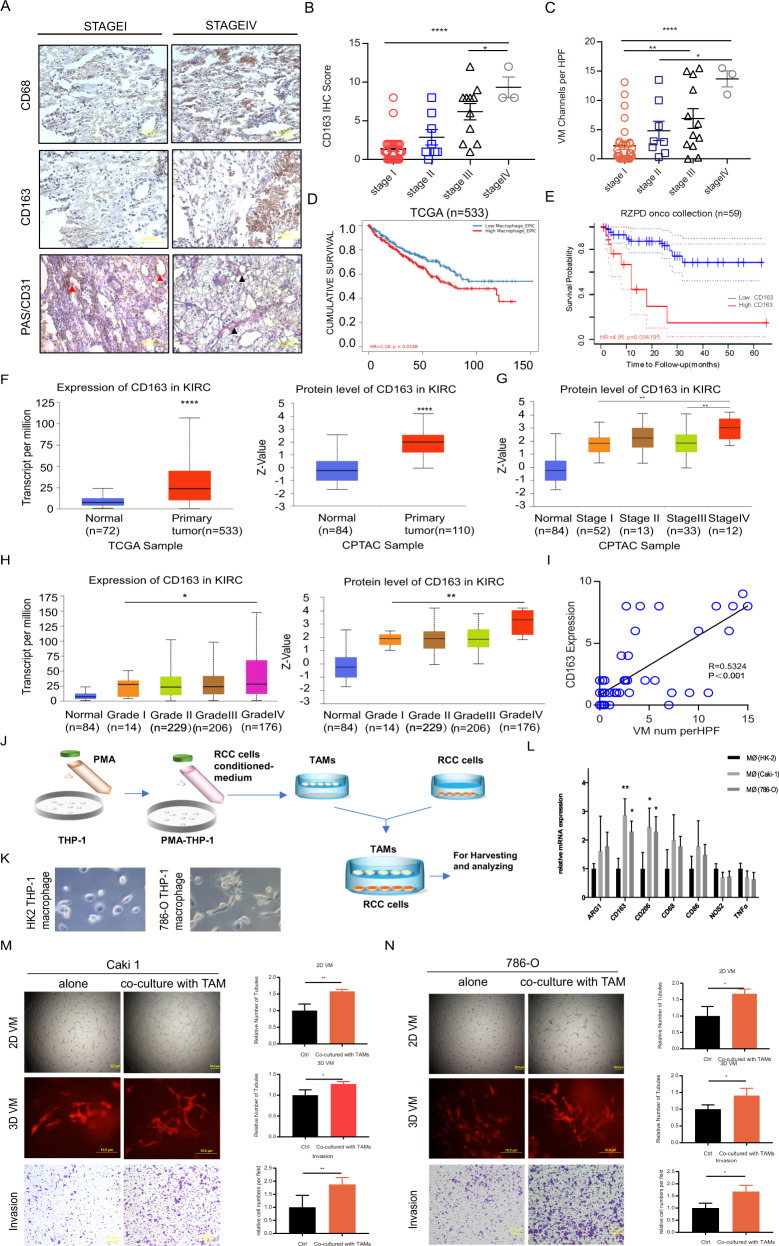
TAMs may influence VM in ccRCC patients. **A** CD31/PAS were utilized to assess VM; CD68 and CD163 were applied to identify M2 macrophages in ccRCC patients at TNM stage I and stage IV. Black arrows indicate the VM channels surrounded by tumor cells with PAS+/CD31−, and red arrows show the endothelial cell-dependent vessels with PAS+/CD31+ staining. **B** The expression of CD163 in ccRCC patients at different clinical stages. **C** The number of VM channels per HPF in ccRCC samples from different clinical stages. **D** The overall survival probability of ccRCC patients was negatively correlated with high macrophage infiltration based on the TCGA database. **E** The overall survival probability of ccRCC patients was negatively correlated with high CD163 expression based on data from The German Cancer Research Center. **F** The expression of CD163 in ccRCC primary tumor samples and adjacent normal tissues at the mRNA level (left) from the TCGA database and at the protein level (right) from the CPTAC database. **G** The protein level of CD163 in CPTAC samples of different stages of ccRCC. **H** The expression of CD163 in clinical samples of different pathological grades of ccRCC at the mRNA level (left) in the TCGA database and at the protein level (right) in the CPTAC database. **I** The correlation between the level of CD163 expression and VM based on analysis of our clinical samples. **J** Schematic diagram of coculture in vitro. **K** THP-1 cells were induced into macrophages with phorbol-12-myristate-13-acetate (PMA) added and then cocultured with macrophages with human kidney cells, HK-2 cells and 786-O cells. Representative images of the two groups (HK-2 coculture with macrophages and 786-O coculture with macrophages) are shown. **L** qRT-PCR was applied to test M1 and M2 biomarkers (CD86, NOS2, TNFα, arginase-1, CD163, CD206, CD68) in macrophages after coculture with Caki-1 cells and 786-O cells. **M**, **N** Matrigel-coated 2D and collagen-based 3D VM formation ability and invasion capacity were measured in Caki-1 and 786-O cells with/without coculture with TAMs. Data are presented as means ± SD. *p < 0.05, **p < 0.01, ***p < 0.001, ****p < 0.0001.

To illuminate the correlation between TAMs and VM in ccRCC, as well as the clinical significance of TAMs, we collected 51 ccRCC human specimens and the corresponding clinicopathological data. To detect TAMs and VM, we performed immunostaining of the M2 macrophage biomarkers CD68/CD163 and implemented PAS/CD31 double staining. The results (Fig. 1A) indicated that both TAM (CD68/CD163 positive) recruitment and VM (PAS positive/CD31 negative) were significantly enhanced in RCC tissues of patients in stage III and stage IV compared with those in stages I and II (Fig. 1B, C). Interestingly, according to the cohort from The Cancer Genome Atlas (TCGA) database, RCC patients with a high level of macrophage infiltration had a lower cumulative survival rate than patients with a low level according to TIMER2.0 [18]. The split infiltration percentage of patients was 50% and the cumulative survival was analyzed by Kaplan-Meier curve parameters. (Fig. 1D). Consistently, based on data from The German Cancer Research Center, RCC patients with a high level of M2 macrophage recruitment had inferior survival (http://dna00.bio.kyutech.ac.jp/PrognoScan/index.html) (Fig. 1E). Moreover, the expression of CD163 in primary tumor tissue was significantly higher than that in adjacent normal tissue at both the mRNA and protein levels in RCC patients based on the TCGA and Clinical Proteomic Tumor Analysis Consortium (CPTAC) databases (Fig. 1F). We compared CD163 protein levels across RCC patients with different clinical stages and found that CD163 expression was significantly higher in stage IV than in earlier stages (Fig. 1G). RCC patients with high-grade disease also had higher mRNA and proteins levels of CD163 than those with low-grade (Fig. 1H). The above analysis revealed that TAMs infiltration was higher in tumor tissues than in adjacent tissues and that the infiltration level increased along with increasing clinical stage. Another TAM marker, CD68, has also been analysed with its expression and prognostic value based on TCGA database and data from The German Cancer Research Center, whose results were consistent with above analysis (SFig. 1A–C). According to the immunohistochemical results from our clinical samples, both CD163 and CD68 expression was positively correlated with VM enhancement, suggesting that the increased recruitment of TAMs was associated with VM enhancement in RCC tumor tissues (Fig. 1I and D).

To further validate positive correlation between TAMs and VM in vitro, we applied a coculture system of TAMs and RCC cells (786-O and Caki-1 cells). As shown in the schematic diagram (Fig. 1J), we first treated THP-1 cells with 100 ng/ml PMA to obtain macrophages and then added CM collected from RCC cells to macrophages. Morphologic images of the induced macrophages were observed for confirmation, which showed that macrophages induced by 786-O cells appear more stretched and elongated than macrophages induced by HK-2. (Fig. 1K). M2 and M1 markers (arginase-1, CD163, CD206, CD68, CD86, NOS2, TNFα) were detected in macrophages treated with Caki-1-CM and 786-O-CM (with HK-2-CM as a control) using quantitative real-time PCR (qRT-PCR). The results revealed that M2 markers such as CD163 and CD206 were significantly increased in TAMs induced by CM from RCC cells compared with induced by HK-2-CM. (Fig. 1L). As expected, coculture with TAMs prominently enhanced both Matrigel-coated 2D and collagen I-based 3D VM, as well as Caki-1 and 786-O cell invasion (Fig. 1M, N).

### TAMs can downregulate TIMP2 expression to promote VM and cell invasion in RCC in vitro

**Fig. 2:**
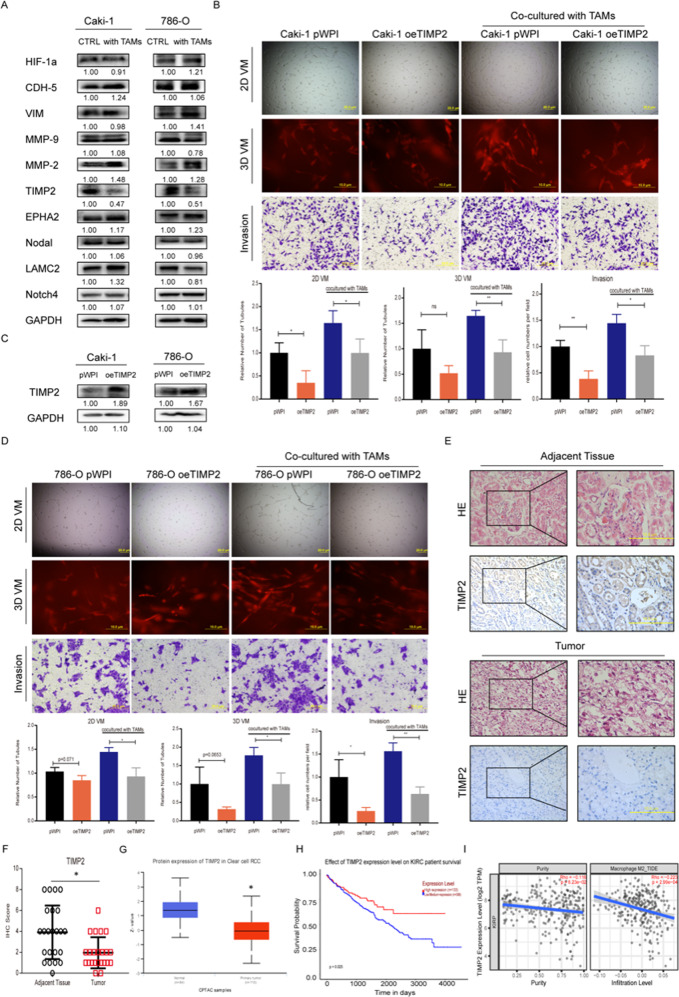
TAMs can downregulate TIMP2 expression to promote VM and cell invasion in RCC in vitro. **A** Western blot assays for the 10 genes related to VM in Caki-1 cells and 786-O cells cocultured with TAMs versus the control. **B** Western blot assay was applied to verify the efficacy of TIMP2 overexpression (oeTIMP2) through transfection with TIMP2-cDNA in Caki-1 cells and 786-O cells. (c-d) 2D/3D VM and Transwell invasion assays were performed in Caki-1 cells (**C**) and 786-O cells (**D**) transfected with TIMP2-cDNA and pWPI control vector after coculture with macrophages for 48 h. **E** Representative H&E and IHC staining of TIMP2 in adjacent noncancerous tissues (above) compared to paired ccRCC tissues (below). **F** The IHC score of TIMP2 between 23 paired adjacent tissues and ccRCC tumors. **G** The protein level of TIMP2 in ccRCC samples (*n* = 110) and normal tissues (*n* = 84) from the CPTAC database. **H** The prognostic value of TIMP2 for predicting the overall survival of ccRCC patients based on the TCGA database. **I** The correlation between TIMP2 expression and M2 macrophage infiltration levels. Data are presented as means ± SD. *p < 0.05, **p < 0.01.

To explain the molecular basis for TAM-mediated VM in RCC cells, we assessed ten VM-related genes [19, 20] via Western blot assay in RCC cells with or without TAM coculture to examine if any of those genes would be responsive to coculturing. The results revealed that TIMP2 expression was only one significantly altered after coculture with TAMs. Comparing all other VM-related genes, we selected TIMP2 as our potential candidate; its expression consistently decreased after coculture with TAMs (Fig. 2A). To validate that TIMP2 is the functional candidate regulated by TAMs to impact VM, we applied rescue experiments. After transfecting Caki-1 and 786-O cells with TIMP2-cDNA (oeTIMP2) versus pWPI-cDNA (Fig. 2B), the tumor cells were collected for 2D/3D VM assays and invasion assays after coculture with/without TAMs. The results showed that oeTIMP2 in RCC cells could partially reverse the increased VM formation induced by coculturing with TAMs (Fig. 2C, D). In contrast, decreasing TIMP2 via adding TIMP2-shRNA led to increase more significantly VM formation and invasion in 786-O cells and Caki-1 cells (Sfig. 2A–D). To demonstrate this macrophage-regulated TIMP2 expression and function only occurred in RCC cells, we further examined TIMP2 expression in HK-2 cells after co-culture with macrophage/TAM and found out that this effect could not exert on normal renal cells (Sfig. 2E).

To further confirm the role of TIMP2 in RCC clinical samples, we detected its expression via immunohistochemistry (IHC) in 23 pairs of human clinical samples derived from ccRCC tumor tissues and adjacent normal tissues. The results showed that TIMP2 was much lower in tumor tissues than in paired adjacent normal tissues (*p* < 0.001; *n* = 23) (Fig. 2E, F), which was consistent with the results extracted from CPTAC protein database (Fig. 2G). Additionally, analyzed by GEPIA2 (http://gepia2.cancer-pku.cn/#index), low expression of TIMP2 led to dramatically worse overall survival of ccRCC patients from TCGA database (Fig. 2H). In addition, we analyzed the correlation between TIMP2 and M2 macrophage infiltration with TIMER2.0 and found a significant positive correlation between them (Fig. 2I).

These findings suggest that TIMP2, restrained by TAMs, may be a critical VM regulator in RCC.

### TAM-derived exosomes promote VM and cell invasion in RCC

**Fig. 3:**
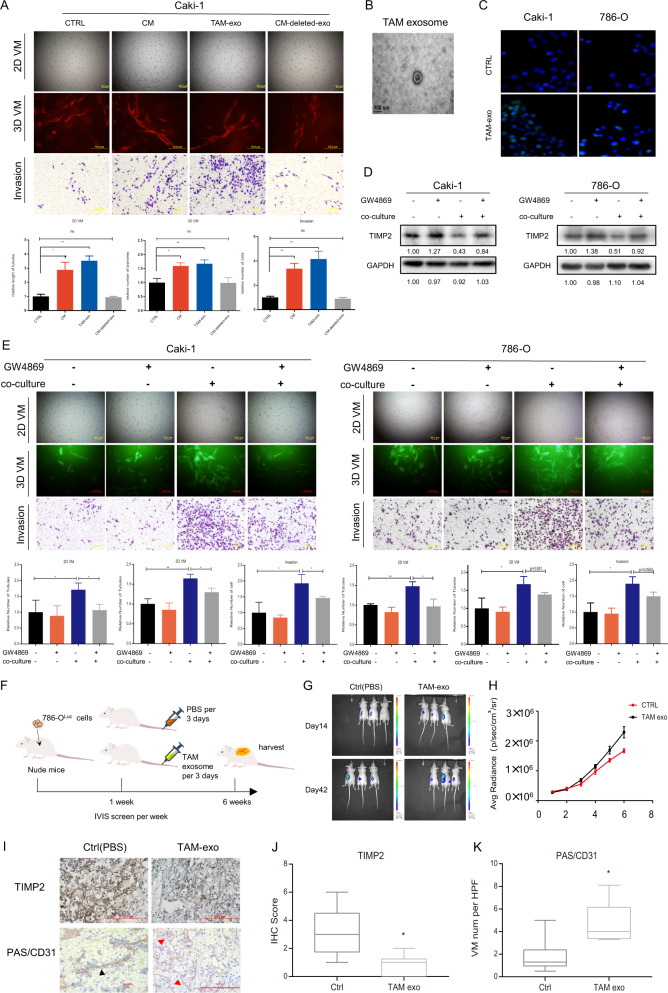
TAM-derived exosomes promote VM and cell invasion in RCC. **A** 2D/3D VM and Transwell invasion assays were performed in Caki-1 cells after adding conditioned medium (CM), TAM-derived exosomes and exosome-removed CM with PBS as a control. **B** Photograph of electron microscopy detection of TAM-derived exosomes. **C** TAM-derived exosomes labeled with DiO were added to Caki-1 and 786-O cells. Fluorescence microscopy was used to detect green signals in RCC cells. **D** Western blotting was used to detect TIMP2 expression in Caki-1 cells (left) and 786-O cells (right) after adding exosome inhibitor (GW4869) to the coculture system. **E** 2D/3D VM formation ability and invasion capacity were measured in Caki-1 (left) and 786-O cells (right) after adding exosome inhibitor (GW4869) to the coculture system. **F** Schematic diagram of the in vivo experiment. 786-O^Luc^ cells were implanted, and TAM-derived exosomes were injected into the caudal veins of nude mice. **G** IVIS images of mice harboring RCC tumors treated with PBS/TAM exosomes (*N* = 6) for 2 weeks and 6 weeks. **H** The average photons for RCC tumors from xenograft mice described above. **I** Representative images of IHC staining for TIMP2 and VM channels (red triangles show PAS+/CD31−tumor cell-dependent vessels) in mice. **J**, **K** Quantification of the relative IHC staining intensity for TIMP2 expression and the percentage of VM channels. Data are presented as means ± SD. *p < 0.05, **p < 0.01.

Since previous research has reported that macrophages can impact progression of RCC *via* exosome-carried miRNAs [21, 22], we estimated whether MDEs exert an effect on TAM-induced VM in RCC. First, we separately added conditioned medium, purified exosomes from TAM and exosome-removed conditioned medium into Caki-1 cells. After treating Caki-1 cells for 48 h, 2D/3D VM and invasion assays were employed to examine treatment impact on RCC cells. We found that both adding conditioned medium and exosomes into RCC cells could increase VM formation and invasion compared with control group, while adding exosome-removed conditioned medium could not elicit this change (Fig. 3A). Transmission electron microscopy was applied to observe the MDEs; they had distinctive bilayer structures and were round in shape, typically with diameters of 80–100 nm (Fig. 3B). To confirm that these exosomes could be internalized by RCC cells, we added PKH67-labeled MDEs to Caki-1 and 786-O cells for further observation by fluorescence microscopy. Compared with control group without MDEs (which presented blue fluorescence alone), RCC cells incubated with PKH67-labeled exosomes presented merged blue and green fluorescence, indicating the uptake of MDEs by RCC cells (Fig. 3C). Next, exosome-specific biomarkers, such as CD63 and CD9, were detected by Western blot analysis to identify our purified exosomes. The exosome inhibitor GW4869 was utilized to block exosome formation and release and was used as a negative control (Sfig. 3A). After coculturing Caki-1 cells with collected MDEs for 48 h, Western blot analysis, 2D/3D VM and invasion assays were employed to examine effect exerted by MDEs on RCC cells. Treatment with MDEs enabled RCC cells to downregulate TIMP2 expression, thus promoting VM and cell invasion (Sfig. 3B, C). Furthermore, the addition of GW4869 to the coculture system deprived macrophages of ability to influence both Caki-1 and 786-O cells, thus indicating it is the macrophage-derived exosomes function to impact RCC cells’ VM formation and invasion. (Fig. 3D, E).

To determine whether TAM-derived exosomes are responsible for tumor progression *via* TIMP2-regulated VM in vivo, we orthotopically implanted 786-O^Luc^ cells into nude mice to establish the mouse model and divided mice into two groups based on different treatments. TAM-derived exosomes were injected into the caudal veins of the mice every 3 days, while PBS-treated mice were used as the control group (Fig. 3F). Six weeks after implantation, mice were evaluated via a noninvasive in vivo imaging system (IVIS) and sacrificed to harvest tumors for IHC staining. The IVIS scan showed stronger bioluminescence signals in the TAM exosome-treated group than in control group, indicating that larger tumors were formed (Fig. 3G, H). Importantly, IHC staining demonstrated that TAM-derived exosomes reduced TIMP2 expression and promoted VM (PAS+/CD31−) (Fig. 3I–K).

### TAMs decrease TIMP2 expression in RCC cells by altering miR-193a-5p

**Fig. 4:**
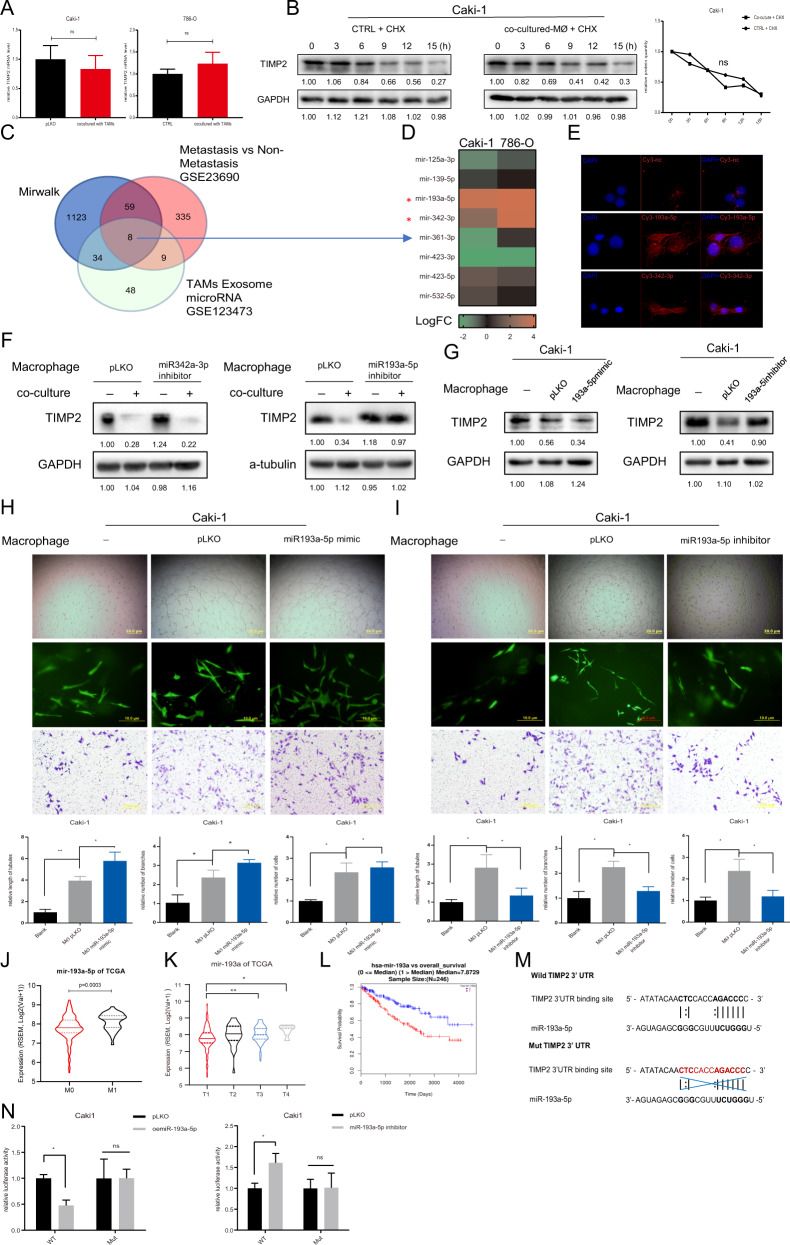
TAMs decrease TIMP2 expression in RCC cells by altering miR-193a-5p. **A** qRT-PCR was used to test TIMP2 mRNA expression in Caki-1 (left) and 786-O cells (right) with/without TAM coculture. **B** Western blotting was used to determine TIMP2 protein stability in Caki-1 cells in the control and coculture groups. **C** Bioinformatics analysis of potential miRNAs present in TAM-derived exosomes and associated with RCC metastasis, as well as predicted to regulate TIMP2 expression. **D** qRT-PCR of 8 potential candidate miRNAs in Caki-1 and 786-O cells cocultured with TAMs compared with the blank control. **E** Fluorescence images of Caki-1 cells were acquired after coculturing with TAMs transfected with Cy3-labeled miR-193a-5p or miR-342a-3p. **F** TIMP2 expression was checked by western blotting after transducing pLKO-miR-342a-3p/pLKO-miR-193a-5p inhibitor into Caki-1 cells in the control and coculture groups. **G** TIMP2 expression in Caki-1 cells was measured after transducing pLKO-miR-193a-5p mimics (left) and pLKO-miR-193a-5p inhibitor (right) into macrophages in a coculture system compared with the blank control. **H** The 2D/3D VM formation ability and invasion capacity of RCC cells were measured after transducing pLKO-miR-193a-5p mimics into macrophages in a coculture system compared with the blank control. **I** The 2D/3D VM formation ability and invasion capacity of RCC cells were measured after transducing pLKO-miR-193a-5p inhibitor into macrophages in a coculture system compared with the blank control. **J** The level of miR-193a-5p in patients without/with metastasis (M0/M1) from the TCGA database. **K** The level of miR-193a in TCGA samples of different clinical stages of ccRCC. **L** The overall survival probability of ccRCC patients was negatively correlated with high miR-193a levels based on the cohort data from the TCGA database. **M** Sequences of WT and MT TIMP2 3′ UTRs were designed. **N** Luciferase reporter activity was measured after transfection of WT or MT TIMP2 3′ UTR in Caki-1 cells with pLKO/oemiR-193a-5p (left) and pLKO/miR-193a-5p inhibitor (right). Data are presented as means ± SD. *p < 0.05, **p < 0.01, ns no significance compared with the control.

Molecular constituents embedded in exosomes play key roles in cell communication in the tumor microenvironment, and miRNAs are dominant exosomal constituents [23]. To clarify the mechanisms by which TAM-derived exosomes foster VM and cell invasion, we first assessed changes in TIMP2 mRNA levels with TAM-derived exosome treatment. As shown in Fig. 4A, there was no significant change in TIMP2 mRNA level between treated and control groups, signifying that transcriptional regulation is an unlikely mechanism. Cycloheximide (CHX), an inhibitor of protein synthesis, was applied to Caki-1 cells to assess protein stability of TIMP2, and the results showed no variation between the coculture group and the blank control, indicating that the mechanism is not mediated by translational regulation (Fig. 4B).

Then, we assessed miRNAs that could bind to the 3′ UTRs of target mRNA to posttranscriptionally regulate TIMP2 expression. By bioinformatic analysis, we screened out a set of potential miRNAs that are capable of targeting TIMP2, are associated with metastasis and are highly expressed in TAM-derived exosomes (Fig. 4C). Further experiments testing the expression of these miRNAs in response to TAM-derived exosomes revealed that both miR-342a-5p and miR-193a-5p increased after TAM-derived exosome treatment in RCC cells (Fig. 4D). We transfected Cy3-labeled miR-193a-5p and miR-342a-5p into macrophages and then cocultured the transfected macrophages with 786-O cells. After 48 h of incubation, Cy3-positive 786-O cells were observed by immunofluorescence, indicating that these two miRNAs were transferred from macrophages into RCC cells (Fig. 4E). The suppression of miR-193a-5p and miR-342a-5p in Caki-1 cells with vector-based miRNA inhibitors revealed that inhibition of miR-193a-5p could reverse the influence of coculture on TIMP2 expression (Fig. 4F). In addition, to better evaluate the effect of miR-193a-5p on TIMP2 expression as well as VM and cell invasion, we altered miR-193a-5p expression in macrophages by adding its mimics or inhibitors for coculture with Caki-1 cells (Fig. 4G). The results showed that miR-193a-5p mimics had a stronger effect than the vector control in facilitating 2D/3D VM and cell invasion with the downregulation of TIMP2 expression in Caki-1 cells. miR-193a-5p inhibitors obviously reversed these effects in the coculture system (Fig. 4G–I). TCGA database analysis showed that the level of miR-193a-5p was higher in patients with metastasis than in patients without metastasis; it was also higher in patients with late-stage RCC (T3/T4) than in those with early-stage RCC (T1) (Fig. 4J, K). In addition, RCC patients with high expression of miR-193a-5p had worse survival than those with low expression (Fig. 4L).

Furthermore, to confirm whether TIMP2 is directly targeted by miR-193a-5p, the WT 3′ UTR or a MUT 3′ UTR with deletion of the miRNA-binding site (Fig. 4M) (predicted by database TargetScan) was cloned into vector psiCHECK2 for luciferase reporter assay. Compared with the control conditions, miR-193a-5p overexpression reduced the luciferase activity of the WT TIMP2 3′ UTR but not the MT TIMP2 3′ UTR. In contrast, inhibiting miR-193a-5p enhanced the luciferase activity of the WT TIMP2 3′’ UTR but not the MT 3’ UTR (Fig. 4N). Overall, we uncovered that exosome-derived miR-193a-5p from macrophages targets TIMP2 in RCC cells to enhance VM and cell invasion capacity.

### TAMs upregulate miR-193a-5p expression via HIF1α-mediated transcriptional modulation

**Fig. 5:**
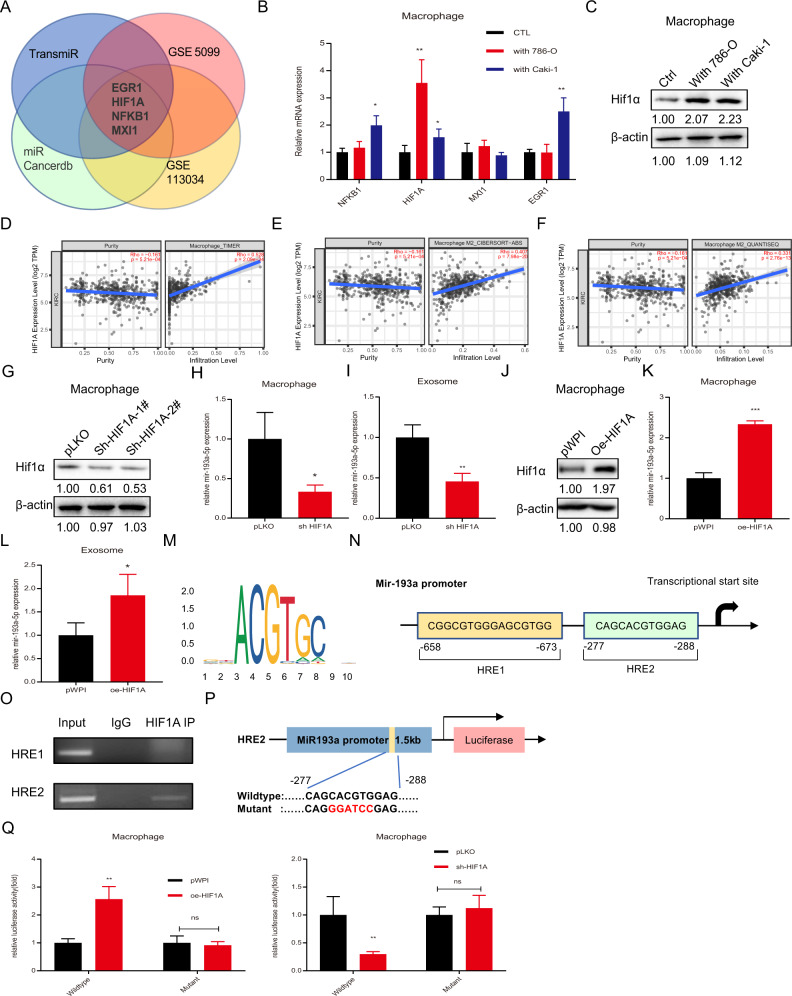
TAMs upregulate miR-193a-5p expression via HIF1α-mediated transcriptional modulation. **A** Venn diagram of bioinformatics analysis to indicate that the potential upstream genes might regulate miR-193a-5p. **B** qRT-PCR was used to determine 4 potential candidate genes in macrophages cocultured with Caki-1 and 786-O cells compared with the blank control. **C** Western blotting was used to verify HIF1α expression in macrophages cocultured with Caki-1 and 786-O cells compared with the blank control. **D** The correlation between HIF1α gene expression and macrophage infiltration level in RCC based on the TCGA database. **E**, **F** The correlation between HIF1α gene expression and M2 macrophage infiltration level in RCC according to different algorithms based on the TCGA database. **G** Western blotting was applied to test the efficacy of knocking down HIF1α by pLKO-shHIF1α-1#/pLKO-shHIF1α-2# in macrophages. **H**, **I** qRT-PCR was used to test the level of miR-193a-5p in macrophages (**H**) and MDEs (**I**) after knocking down HIF1α expression in macrophages. **J** Western blotting was applied to measure the efficacy of HIF1α overexpression by pWPI-oeHIF1α. **K**, **L** qRT-PCR showed the level of miR-193a-5p in macrophages (**K**) and MDEs (**L**) after overexpressing HIF1α in macrophages. **M** HRE motif sequence identified with JASPAR. **N** The structure of the HRE binding site in the 3-kb miR-193a promoter region. **O** ChIP assay confirmed that HIF1α could directly bind with miR-193a HRE2 (−277 nt to −288 nt). **P** Schematic diagram of WT and MT pGL3-miR-193a promoter-reporter constructs. **Q** The luciferase reporter assay was performed to detect promoter activity after transfection of HRE WT or MT miR-193a promoter pGL3-luciferase plasmids into macrophages with pLKO or shHIF1α. Data are presented as means ± SD. *p < 0.05, **p < 0.01, ***p < 0.001, ns no significance compared with the control.

To dissect the mechanism by which TAMs upregulate miR-193a-5p, we performed bioinformatic analysis of online databases and predicted four candidate genes (EGR1, HIF1A, NFKB1 and MXI1) that might transcriptionally regulate target miRNA expression (Fig. 5A). To screen for potential transcription factors of miR-193a-5p, we measured the mRNA levels of the four candidates in macrophages cocultured with RCC cells (versus macrophages alone as a control). We found that HIF1A was significantly increased in both 786-O and Caki-1 coculture groups (Fig. 5B). The protein level of HIF-1α also prominently increased after RCC cell coculture (Fig. 5C). A search of TIMER2.0 [24] revealed a significant positive correlation between HIF1A and macrophage infiltration as well as HIF1A and M2 macrophage infiltration in RCC (Fig. 5D–F). Moreover, after knocking down the expression of HIF-1α, the level of miR-193a-5p was decreased not only in macrophages but also in MDEs (Fig. 5G–I). Correspondingly, the miR-193a-5p level was elevated due to HIF-1α overexpression (Fig. 5J–L).

To explore whether HIF-1α directly activates the transcription of miR-193a, we predicted HIF-1α-responsive elements (HREs) in the 2-kb region of the miR-193a promoter by searching the JASPAR database and selected two rigorously screened HREs (HRE1 − 658 nt to −673 nt, HRE2 − 277 nt to −288 nt) (Fig. 5M, N). A chromatin immunoprecipitation (ChIP) assay was performed to illustrate that HIF-1α could successfully bind to HRE2 on the miR-193a promoter region to initiate its transcription (Fig. 5O). Based on the ChIP results, we constructed a pGL3-luciferase plasmid including the WT miR-193a promoter or the MT promoter, which had mutations in the key sequence of HRE2 (Fig. 5P). Following transfection of the constructed plasmids into macrophages with different treatments, the luciferase activities were evaluated. As expected, HIF-1α overexpression enhanced the luciferase activity in macrophages transfected with the WT promoter but not in macrophages transfected with the MT promoter. In return, knocking down HIF-1α expression significantly reduced luciferase activity in macrophages transfected with the WT reporter but not in macrophages transfected with the MT reporter (Fig. 5Q).

### A preclinical study using an in vivo mouse model revealed that inhibition of miR-193a-5p in MDEs represses tumor progression and metastasis

**Fig. 6:**
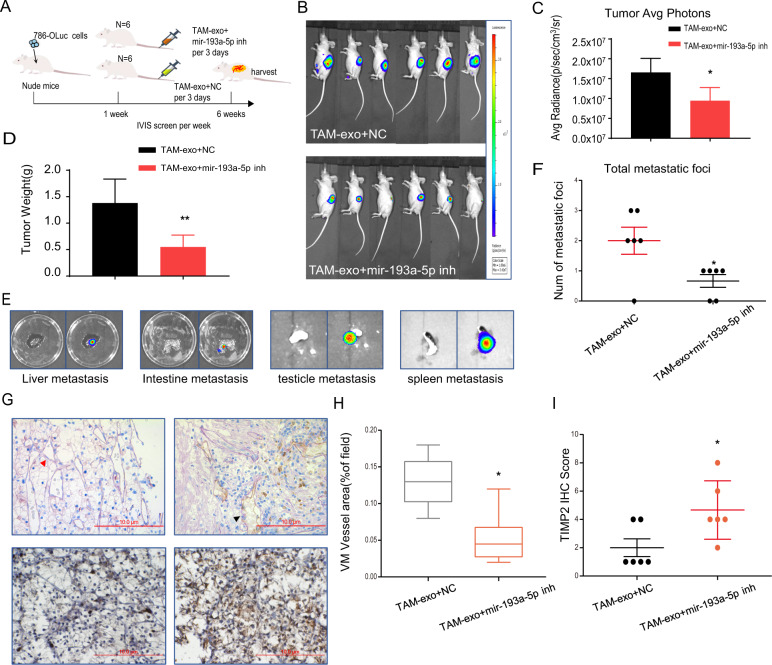
Preclinical study using an in vivo mouse model revealed that inhibition of miR-193a-5p in MDEs represses tumor progression and metastasis. **A** A sketch map of tumor formation in nude mice. Macrophages were transfected with NC miRNA inhibitor or miR-193a-5p inhibitor, after which exosomes were extracted and injected into nude mice (*N* = 6). **B** IVIS images of mice harboring RCC tumors after orthotopic implantation of 786-O cells for 6 weeks. **C** Tumor average photons for ccRCC from xenograft mice. **D** Tumor volume in each group was observed and measured after mice were sacrificed. **E** Representative organ bioluminescent images showing metastasis from the liver, intestine, testicle, and spleen. **F** Quantification of the total metastatic foci. **G** Representative images of IHC staining for TIMP2 and VM vessel area (red triangles show PAS+/CD31−tumor cell-dependent vessels) in mice. **H** Evaluation of area percentage of VM vessels. **I** Quantification of the relative IHC staining intensity for TIMP2 expression. The data are shown as the mean ± SD. *p < 0.05, **p < 0.01, ***p < 0.001, and ns no significance compared with the control.

Exosomes collected from TAMs transfected with NC miR-inhibitor or miR-193a-5p inhibitor were injected into mice that developed ccRCC (Fig. 6A). An orthotopic xenograft mouse model was established by implantation of 786-O^Luc^ cells into nude mice. After 6 weeks, evaluation of IVIS images showed that mice injected with exosomes from TAMs transfected with miR-193a-5p inhibitor showed less bioluminescence than those transfected with NC miR-inhibitor (Fig. 6B, C). In addition, tumor weight and metastatic lesions were detected and measured after the mice were sacrificed. We found that the tumor weight of mice injected with exosomes from miR-193a-5p inhibitor-treated macrophages was significantly decreased compared with that of those injected with exosomes from NC-TAMs (Fig. 6D). The NC-TAM exosome group also developed more metastases (as shown in the liver, intestine, testicle, and spleen) than the miR-193a-5p inhibitor group (Fig. 6E, F). The results of IHC staining validated that TAM-derived exosomes weakened TIMP2 expression and increased the VM-positive area (PAS+/CD31−), while treating TAMs with a miR-193a-5p inhibitor partially blocked the function of TAM-derived exosomes (Fig. 6G–I).

Together, the results from an in vivo mouse study indicated that inhibiting miR-193a-5p levels in TAM exosomes can suppress ccRCC tumor progression and metastasis by suppressing VM and upregulating TIMP2 expression.

## Discussion

**Fig. 7:**
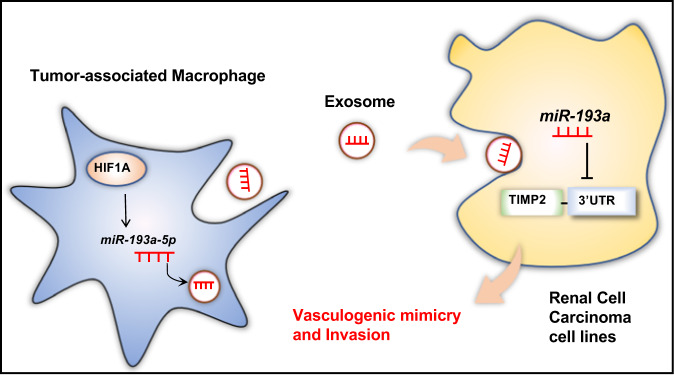
Schematic diagram of how miR-193a-5p shuttled by TAM-derived exosomes downregulates TIMP2 expression in RCC cells to facilitate VM and invasion. The schematic diagram shows the regulatory mechanism how TAM-derived exosomes downregulate TIMP2 expression in RCC cells. The miR-193a-5p upregulated by HIF1A can be shuttled and secreted by TAM-derived exosomes to RCC cells. This TAM-derived exosomal miR-193a-5p downregulates TIMP2 expression to facilitate VM and invasion of RCC cells.

Growing evidence has suggested that TAMs are one of the major populations of the TME and enable immunosuppression, tumor progression, metastasis and therapeutic resistance [25–27]. In this study, we uncovered that M2-type TAMs could promote RCC tumor cell VM and invasion by secreting exosomes. In addition to cytokine-mediated interaction between tumor cells and immune cells, exosome shuttling has also received increasing attention due to its powerful function in TME communication and remodeling [28–30]. Our findings validated the critical action played by TAM-derived exosomes in TAM-mediated tumor cell VM and elucidated the underlying regulatory mechanisms. Together with other studies [21, 31], we have demonstrated that miRNAs loaded in exosomes are functionally required for cell-cell communication and interplay in the TME. According to the mechanistic clarification steps of our study (Fig. 7), miR-193a-5p enveloped in TAM-derived exosomes encourages RCC cell VM by specifically sponging the 3′ UTR of TIMP2. Additionally, our in vivo experiment proved the efficacy of TAM-derived exosome-secreted miR-193a-5p in boosting tumor growth and metastasis. With further study of the biofunctions of exosome-shuttled molecules, we can better understand cell-to-cell communication in the TME and design molecule-targeted remedies for improving diagnostic approaches and treatments for tumors.

Although abundant studies have disclosed that TAMs can facilitate tumor cell proliferation, invasion and migration, whether TAMs could encourage tumor cell VM has not been clearly illustrated. Numerous emerging papers studying VM have provided us with a better understanding of the role of VM in tumor development and treatment [13, 32]. Our present study analyzed clinical samples from RCC patients and found that CD163-positive TAM recruitment was positively correlated with VM occurrence. Consistently, TAM recruitment and VM were significantly enhanced in later stages. Analysis of the association between macrophages or CD163-positive TAMs and clinical outcome confirmed their unfavorable prognostic value in ccRCC. Previous clinical research also revealed that VM is an independent adverse prognostic feature for ccRCC patients [33]. All these findings provide strong evidence for our further investigation on the interaction between TAMs and VM in RCC cells. In this scenario, we mainly explored how TAMs support tumor cells progression and assessed the detailed mechanisms by which TAM-derived exosomes mediate this process of cellular communication. Additionally, in support of previous studies, we demonstrated that CM collected from RCC cells could successfully induce M2 macrophage polarization. Notably, data from the existing research signified that the crosstalk between tumor cells and TAMs boosts the protumor characteristics of TAMs in the TME [29, 34]. However, we only examined how exosomes secreted from TAMs affect the malignancy of tumor cells and assessed the underlying molecular mechanisms. The impact of tumor cells on macrophages and the involved mechanisms remain to be further probed. Investigation of how tumor cells educate macrophages and of the regulatory loop driven by their interaction could lead better treatment options.

In addition to the influence of VM on tumor progression and metastasis, the effect of VM on the response to antiangiogenic agents seem to be noteworthy. A study on breast cancer reported that the antiangiogenic agent sunitinib, a VEGF receptor tyrosine kinase inhibitor (TKI), could facilitate VM by inducing intratumoral hypoxia. Moreover, VM in turn facilitated tumor metastasis and sunitinib resistance [35]. Coincidentally, it has been reported and verified that VM induced by sunitinib treatment could ultimately lead to resistance to antiangiogenic therapies in ccRCC [17]. Interestingly, in addition to the promotive role of macrophages in therapeutic drug resistance in cancer [36], macrophages can also decrease the efficacy of antiangiogenic therapies [37]. The previous findings and our current findings suggest that targeting macrophages in RCC would not only suppress VM to slow progression and metastasis but also help to prevent tumor cell resistance to antiangiogenic agents. Thus, based on the results of this study, further exploration of the impact of TKI resistance in RCC exerted by macrophage induced VM may provide a promising strategy for combination therapy to overcome the unsatisfactory curative outcomes of current clinical therapeutic strategies.

In conclusion, exosome-shuttled miR-193a-5p is transmitted by TAMs to promote VM and cell invasion in RCC, and this newly identified regulatory pathway can be exploited as a potential therapeutic target to better suppress RCC progression.

## Materials and methods

### Patients and samples

A total of 51 histologically confirmed clear cell renal cell carcinoma (ccRCC) tissue samples with 23 paired adjacent noncancerous tissues were obtained between August 1st, 2014, and February 1st, 2016, from the Department of Urology, the Second Affiliated Hospital of Harbin Medical University (Harbin, China). Patients were removed if they had been treated with previous neoadjuvant chemotherapy or receptor tyrosine kinase inhibitors (RTKis). All samples collected for use in research after patients signed the Scientific Ethics Consent were fixed in 10% formalin and then embedded in paraffin. Our research was approved by the institutional review board of the hospital in advance. Patients information are listed in Supplementary Table 1.

### Cell culture

The human RCC cell lines 786-O and Caki-1 and the monocytic leukemia cell line THP-1 and HEK 293 T were purchased from the American Type Culture Collection (ATCC, Manassas, VA). Caki-1, 786-O and HEK 293 T cells were cultured in DMEM. THP-1 cells were cultured in RPMI-1640 medium. Both supplemented with 10% fetal bovine serum (FBS), antibiotics (100 units/ml penicillin, 100 mg/ml streptomycin), and 2 mM glutamine (Invitrogen, Grand Island, NY, USA). All cell lines were maintained in a humidified 5% CO_2_ environment at 37 °C.

### Inducing macrophage from monocyte

THP-1 cells were cultured with 100 ng/ml phorbol 12-myristate 13-acetate, PMA (Sigma, St Louis, MO, USA) in normal RPMI-1640 medium with 10% exosome-free FBS for 48 h to allow differentiation into macrophages. Then THP-1-induced macrophages were refreshed with medium without PMA for 2 days before use.

### Coculture experiment

Macrophages were seeded in Transwell chambers with 0.4-μm pores (Corning Life Science, NY, USA), and RCC cells were seeded in the bottom of a 6-well plate. After 2 days of coculture, RCC cells and conditioned medium (CM) were harvested for further experiments. Exosome secretion inhibitor GW4869 (MedChemExpress, Monmouth Junction, NJ, USA) was employed for CM treatment at 5 μM concentration. DMSO (Sigma) was used as the control for GW4869.

### Exosome isolation and identification

The cultured medium was harvested and centrifuged at 1300 rpm for 10 min to collect the supernatant. Then, the supernatant was filtered through a 0.45-µm filtered and centrifuged at 9600 rpm for 20 min; that resulting supernatant was filtered through a 0.2-μm filter to remove residual cells and debris and finally ultracentrifuged at 25800 rpm for 70 min (Beckman Coulter) to collect the pellet containing exosomes and contaminating proteins. To obtain pure exosomes, the pellet was washed with 20 ml of sterile PBS, and ultracentrifugation step was repeated. All centrifugation steps were performed at 4 °C. The exosomes in the pellet were resuspended in PBS or in cell lysis buffer for protein extraction for later experiments. For verification, exosomes were photographed using electron microscopy. Electron micrographs were obtained using a JEOL JEM-1230 (Tokyo, Japan) transmission electron microscope at an 80-kV excitation voltage. Western blot analysis was applied to detect the markers of exosomes (CD9 and CD63).

### Lentivirus packaging and transfection

The lentiviral vector pLKO.1 was used to construct shRNA plasmids, and pWPI was used for overexpression. Both vectors were cotransfected into HEK293T cells with the packaging plasmid psPAX2 and envelope plasmid pMD2.G for 48 h following the standard CaCl_2_ transfection method to produce lentivirus particles dissolved in medium, which were filtered and then used immediately or stored at −80 °C for later use.

### Protein extraction and western blotting

Cells were lysed in cell lysis buffer (RIPA buffer supplemented with phenylmethylsulfonyl fluoride (PMSF) (1:1000) and cocktail (1:100)), and equal amounts of protein samples (30–50 μg) were equally mixed with loading buffer and then boiled and separated on a 6–10% SDS/PAGE gel. Then, the proteins were transferred onto PVDF membranes (Millipore, Billerica, MA), which were blocked with 5% skimmed milk (dissolved in TBST solution) for 1 h before they were incubated with specific primary antibodies overnight followed by HRP-conjugated secondary antibodies for 1 h. An ECL system (Thermo Fisher Scientific) was used for visualization. Primary antibodies used in the study for western blot are listed in Supplementary Table 2.

### RNA extraction and quantitative real-time PCR analysis

Total RNA was extracted using TRIzol reagent (Invitrogen, Grand Island, NY). Superscript III transcriptase (Invitrogen, Grand Island, NY) was applied to reverse transcribe 2 μg of total RNA. qRT-PCR using a Bio-Rad CFX96 system with SYBR green was conducted to determine the mRNA level of a gene of interest, whose expression level was normalized to that of GAPDH (mRNA) or U6 (miRNA) using the 2^−ΔΔ^Ct method. The primers we used for PCR are listed in Supplementary Table 3.

### 2D Matrigel-based tube formation assay

After melting at 4 °C overnight, growth factor-reduced Matrigel (BD Biosciences, USA) was added to 96-well plates at 50 µl per pore and then incubated at 37 °C for one and a half hours. Later, the cells were resuspended in serum-free DMEM at a final concentration of 2.5 × 10^4^ cells/100 µl and added to the wells paved with Matrigel. After 4-6 h of incubation at 37 °C, tube formation was observed using microscopy (Olympus, Tokyo, Japan). Tubules were photographed in 3–5 random fields in each well, and the average length was calculated by ImageJ software according to a previous study [38].

### 3D Collagen 1-induced tube formation assay

Cells suspended in DMEM at the final concentration were added to soluble rat tail type I collagen in acetic acid (Corning, Corning, NY) mixed with 10x reconstitution buffer, 1:1 (v/v) and 1 M NaOH (pH 7, 10–20 μl) [39]. Then, 200 μl of the mixture was loaded into a 48-well culture plate and placed in a cell incubator at 37 °C for 5–7 days.

### Cell invasion assay

Transwell chambers (8-μm pore size) (Corning Life Science) were used for the cell invasion assay. The upper chamber was filled with 100 μl of diluted Matrigel (1:10 dilution) and incubated for 2 h at 37 °C. Then, 150 μl of serum-free medium containing 5 × 104 RCC cells were added to the upper chamber, while 750 μl of the medium containing 10% FBS was added to the lower chamber. Then, methanol and 0.3% crystal violet were used for fixation and staining. The invaded cells were observed and counted under a microscope after removing the noninvasive cells.

### Chromatin immunoprecipitation

Briefly, cell lysates were precleaned with protein A-agarose conjugated normal IgG (sc-2027, Santa Cruz); anti-HIF1A antibody (2.0 µg) (Cell Signaling Technology) was then added to the cell lysates overnight at 4 °C, while IgG was applied as the negative control. Specific primer sets designed for amplifying the target sequence within the miR-193a promoter were used for qPCR. The PCR product was analyzed by agarose gel electrophoresis.

### Luciferase reporter assay

The miR-193a promoter was cloned into the PGL3 basic vector (Promega). After mutating the HIF1A binding site at the promoter region, we cloned the mutant promoter PGL3 vector. The internal control pRL-TK was used in the baseline response. Cells were plated in 24-well plates and transfected with cDNA using Lipofectamine 3000 (Invitrogen, Carlsbad, CA) according to the manufacturer’s instructions. After 36–48 h of transfection, luciferase activity was measured by dual-luciferase assay (Promega). The 3’ UTR of TIMP2 with wild-type (WT) or mutant (MT) miRNA-responsive elements was inserted into the psiCHECK-2 vector (Promega). Cells were plated in 24-well plates and transfected with the constructed cDNA without pRL-TK according to the methods shown above.

### In vivo studies

Athymic BALB/c nude mice (6–8 weeks old) were obtained from Beijing Vital River Laboratory Animal Technology Co., Ltd. Luciferase- labeled 786-O (786-O^-Luc^) cells (2 × 10^6^) were injected into the left renal capsule of male athymic nude mice. After 1 week of inoculation, the nude mice were then divided into different treatment groups. In part 1 animal study, 12 mice were evenly divided into 786-O^-Luc^ + PBS group (injection of PBS into mice tail caudal vein) and 786-O^-Luc^ + TAM-Exo group (injection of TAM-derived exosome into mice tail caudal vein). Tumor growth and metastasis were monitored weekly via IVIS analysis, then counted for statistical analysis. Mice were sacrificed after six weeks since inoculation, and tumors were removed and measured.

In part 2 animal study, the nude mice were randomly divided into two groups (6 in each group). One group was injected with exosomes extracted from macrophages transfected with miR-193a-5p inhibitor into mice tail caudal vein, another group treated with exosomes from macrophages transfected with NC (miRNA inhibitor). Tumor growth and metastasis were monitored weekly via IVIS analysis. Six weeks later, tumors and metastases were obtained from the sacrificed mice, and counted for statistical analysis. All mice were maintained under pathogen-free conditions in the animal facility at Harbin Medical University (Harbin, China). All animal experiments were approved and supervised by the Harbin Medical University Institutional Animal Use and Care Committee. The metastasis information of mice are listed in Supplementary Table 4.

### Immunohistochemistry staining

The procedure was carried out according to the routine manual created by our team previously [40]. After tissue sample preparation, fixation, deparaffinization, hydration, antigen retrieval and blocking, the slices were incubated with primary antibodies in 3% BSA resolved in PBS at 4 °C overnight, followed by biotinylated secondary antibodies (Vector Laboratories, Burlingame, CA, USA). The VECTASTAIN ABC peroxidase system and 3,3’-diaminobenzidine (DAB) kit (Vector Laboratories, Burlingame, CA, USA) were used for visualization. The primary antibodies used were as follows: anti-CD163 (M2 macrophage marker; Thermo Fisher Scientific, 16646-1-AP), anti-F4/80 (total mouse macrophage marker; Abcam, ab16911), and anti-CD31 (JC/70A) (Thermo Fisher Scientific, MA5-13188). Periodic acid Schiff (PAS) reagent (Sigma, 3952016) and prepared 0.5% periodic acid solution (Sigma, 395132) were used for PAS staining. Brown staining characterized positive expression of the target protein. Channels surrounded by tumor cells with PAS + /CD31 − staining represented VM formation. The percentage of positive cells was rated per high-power field (HPF) by 400× magnification as follows: 0 for sections with 1% positive cells; 1 for 1 to 25% positive cells; 2 for 26 to 50% positive cells; 3 for 51% to 75% positive cells; 4 for 76% to 100% positive cells. The staining intensity was graded as follows: weak intensity graded as 1, moderate intensity as 2, and high intensity as 3. Points for the percentage of positive cells and staining intensity were multiplied. Tumor specimens were classified into 3 groups according to overall scoring: negative expression as 0–1, weak expression as 2–4, and high expression as 6–12 points. Total scores were as follows: 0–4 (low) and 6–12 (high). All slides were evaluated independently by 2 pathologists without knowledge of the identity of patients and the clinical outcome.

### Statistics

The experiments were repeated independently at least three times. The results are shown as the mean ± SD. Statistical significance was determined using Student’s t-test and two-way ANOVA with SPSS 22 (IBM Corp., Armonk, NY) or GraphPad Prism 6 (GraphPad Software, Inc., La Jolla, CA). Differences with P values less than 0.05 were considered statistically significant (*p < 0.05, **p < 0.01, ***p < 0.001).

## Supplementary information

supplement data

Original western blot

aj-checklist

## Data Availability

The datasets used and analyzed during the current study are available from the corresponding author on reasonable request.
