# Correction to: Tumor-associated macrophage-derived exosomes transmitting miR-193a-5p promote the progression of renal cell carcinoma via TIMP2-dependent vasculogenic mimicry

**DOI:** 10.1038/s41419-022-05013-2

**Published:** 2022-08-08

**Authors:** Qing Liu, Enyang Zhao, Bo Geng, Shan Gao, Hongyang Yu, Xinyang He, Xuedong Li, Guanglu Dong, Bosen You

**Affiliations:** 1 Department of Radiation Oncology, The Second Affiliated Hospital of Harbin Medical University, 150001 Harbin, China; 2 Future Medical Laboratory, The Second Affiliated Hospital of Harbin Medical University, 150001 Harbin, China; 3 Department of Urology, The Second Affiliated Hospital of Harbin Medical University, 150001 Harbin, China; 4 Department of Pathology, The Second Affiliated Hospital of Harbin Medical University, 150001 Harbin, China

**Keywords:** Tumour angiogenesis, Cancer therapy, Non-coding RNAs

Correction to: *Cell Death and Disease* 10.1038/s41419-022-04814-9, published online 20 April 2022

The original version of this article unfortunately contained an error in figure 2E. The authors accidentally misplaced the HE-stained high magnification image in figure 2E as the IHC image during the last typesetting process. The corrected figure can be found below. The original article has been corrected.
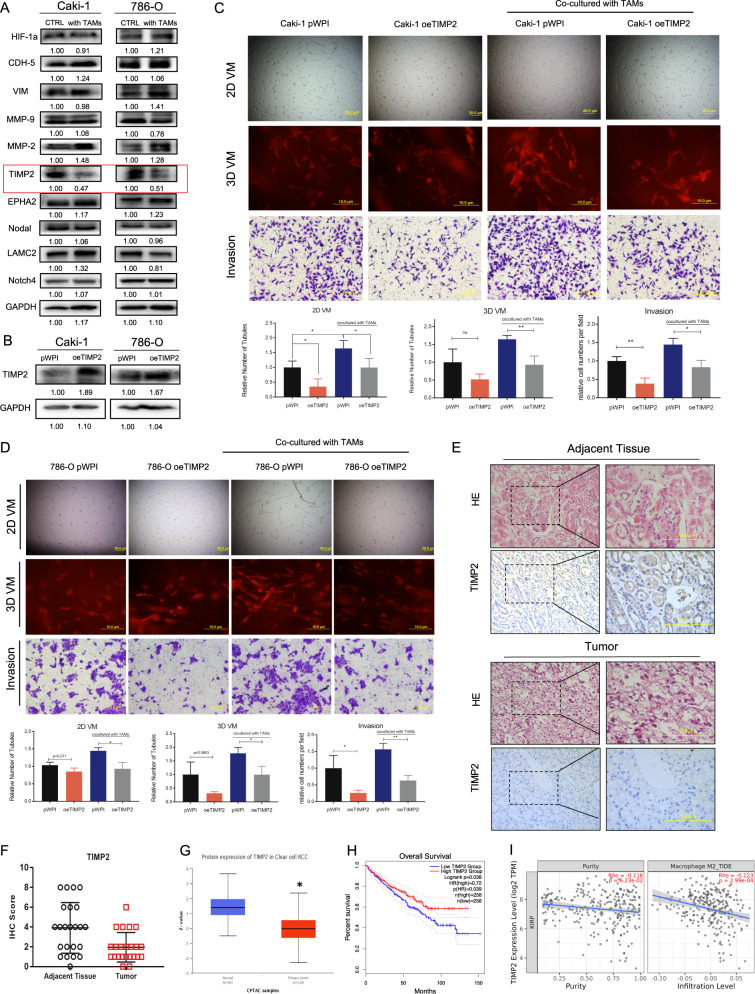


Upon reviewing our raw data, we discovered misplacement of images in Fig. 1A and Fig. 2D, specifically involving three images. This misplacement does not affect the experimental results or the overall conclusions of the article.

