# Correction: Correction to: Tumor-associated macrophage-derived exosomes transmitting miR-193a-5p promote the progression of renal cell carcinoma via TIMP2-dependent vasculogenic mimicry

**DOI:** 10.1038/s41419-025-08075-0

**Published:** 2025-12-23

**Authors:** Qing Liu, Enyang Zhao, Bo Geng, Shan Gao, Hongyang Yu, Xinyang He, Xuedong Li, Guanglu Dong, Bosen You

**Affiliations:** 1 Department of Radiation Oncology, The Second Affiliated Hospital of Harbin Medical University, Harbin, China; 2 Future Medical Laboratory, The Second Affiliated Hospital of Harbin Medical University, Harbin, China; 3 Department of Urology, The Second Affiliated Hospital of Harbin Medical University, Harbin, China; 4 Department of Pathology, The Second Affiliated Hospital of Harbin Medical University, Harbin, China

**Keywords:** Tumour angiogenesis, Cancer therapy, Non-coding RNAs

Correction to: *Cell Death & Disease* 10.1038/s41419-022-05013-2, published online 08 Aug 2022

Upon reviewing our raw data, we discoverd misplacement of images in Fig. 1A and Fig. 2D., specifically involving three images. This misplacement does not affect the experimental results or the overall conclusion of the article.

**original (uncorrected) Fig 1** 
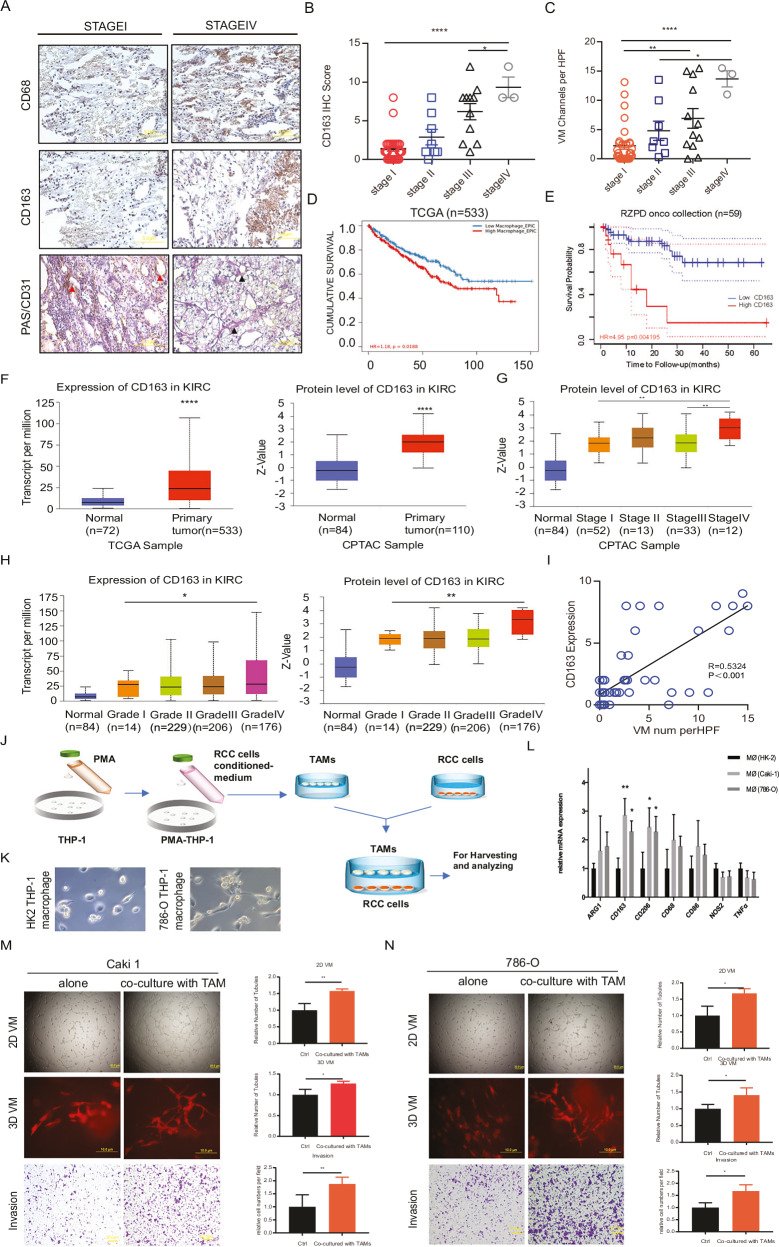


**Amended file of figure 1** 
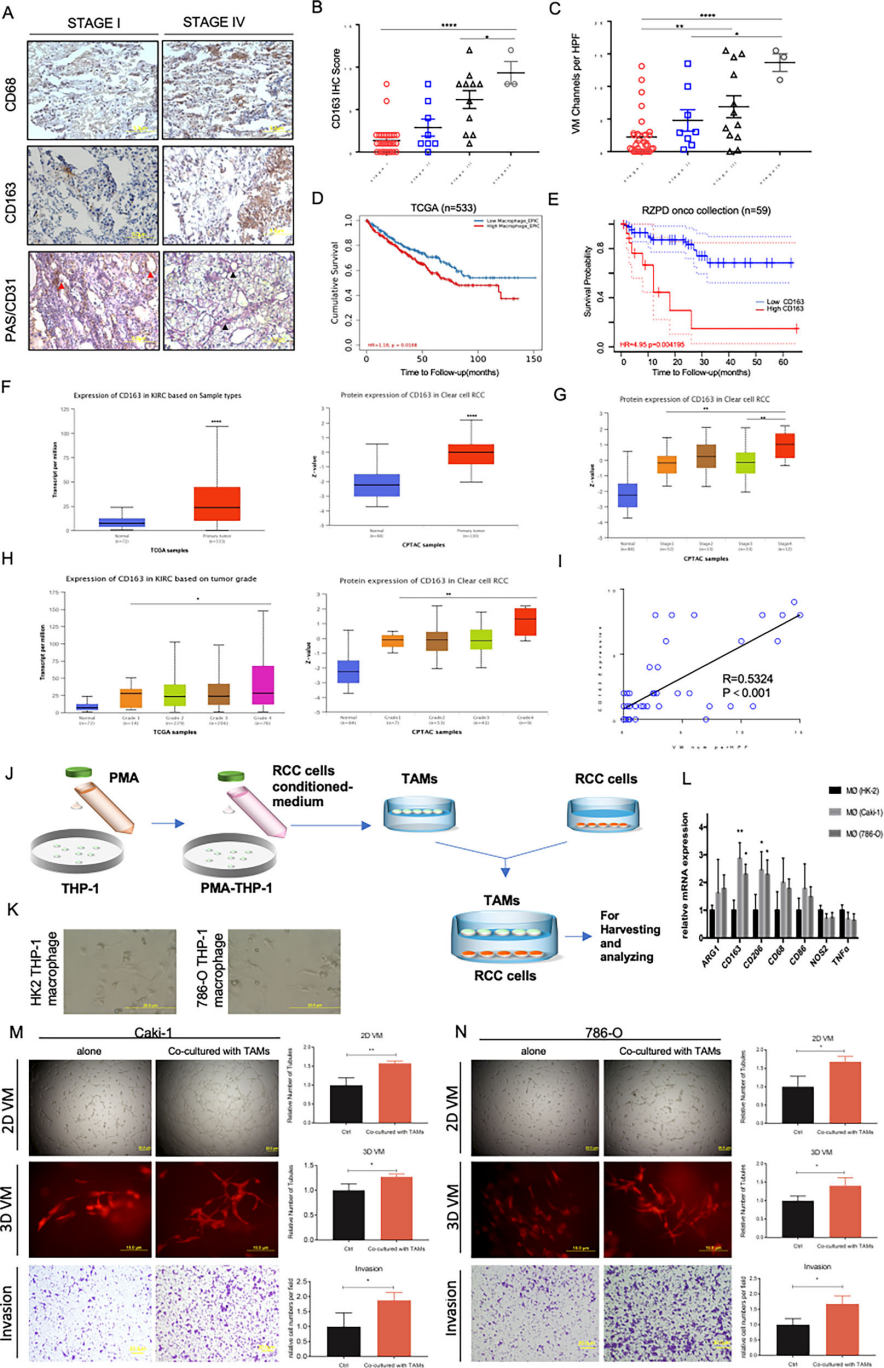


**Original (uncorrected) Fig 2** 
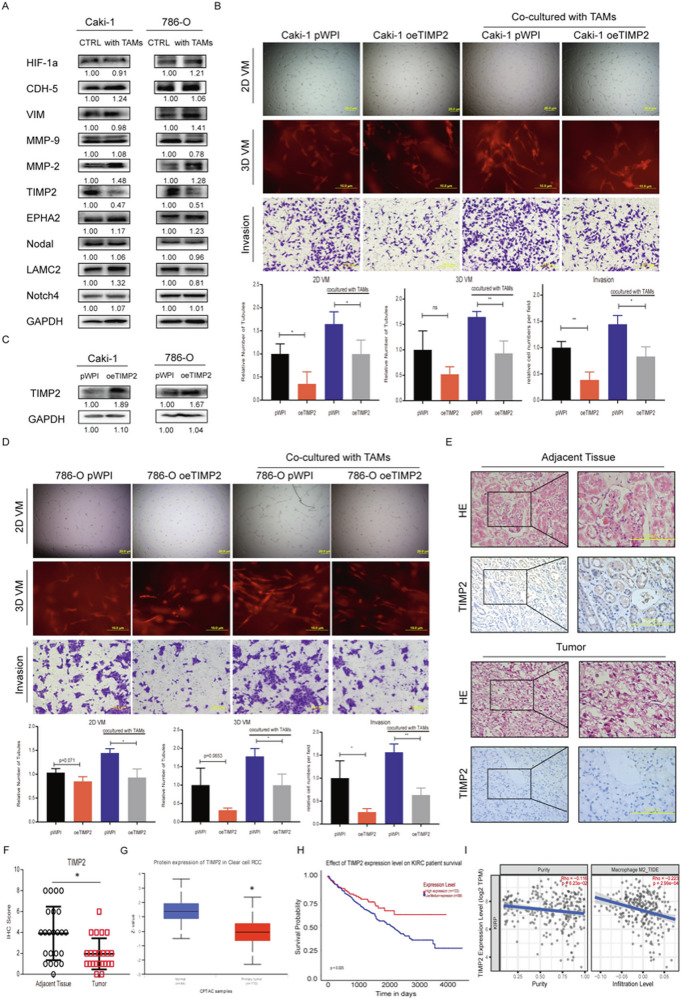


**Amended file of figure 2** 
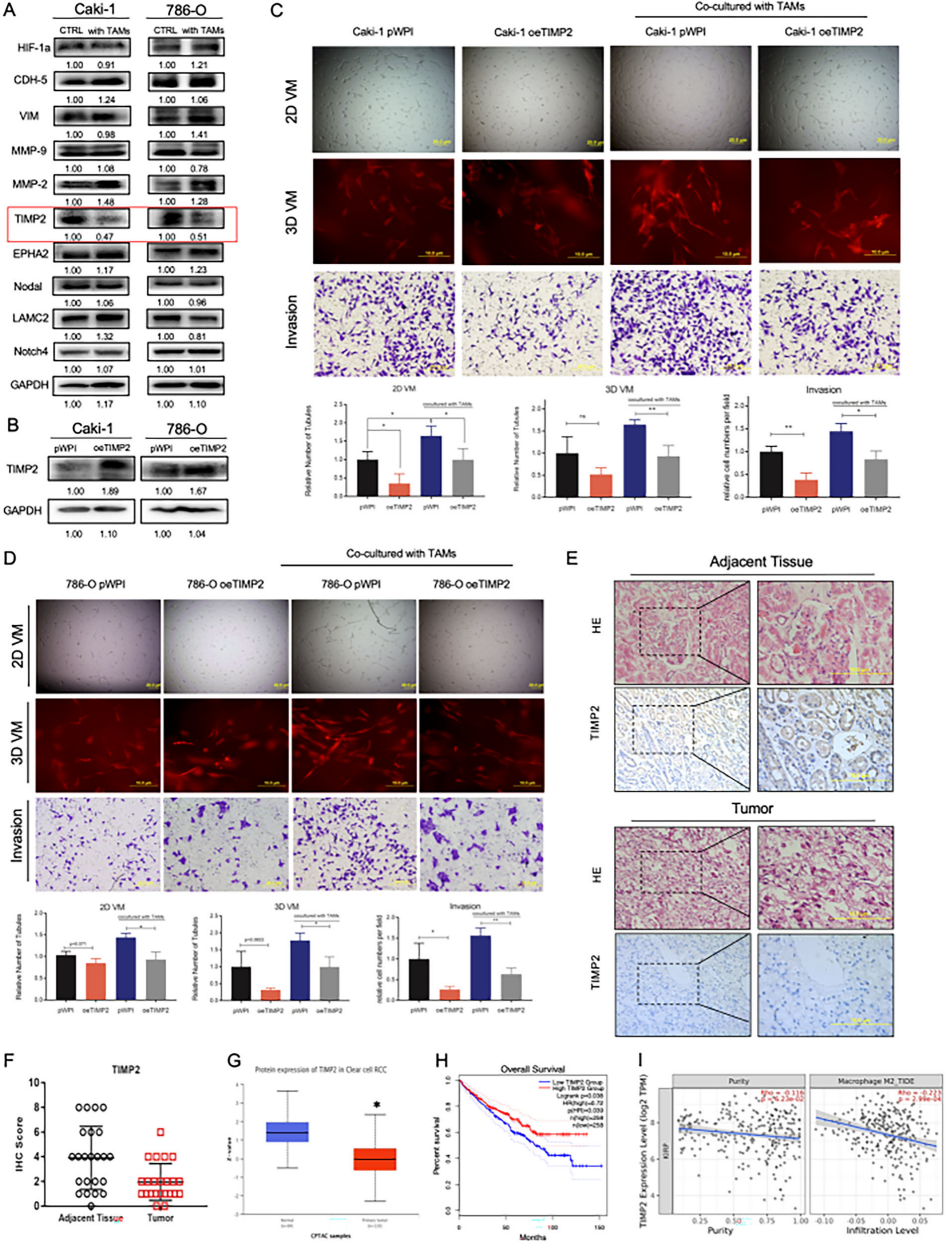


The original article has been corrected.

## Supplementary information

original data of IHC image of CD163 in Fig1A

original data of invasion 786-O pwpi coM in fig2D

original data of invasion 786-O pwpi in fig2D

