# Supplementary material for: Tumor-associated macrophage-derived exosomes transmitting miR-193a-5p promote the progression of renal cell carcinoma via TIMP2-dependent vasculogenic mimicry
Source: Cell Death Dis. 2022 Apr 20;13(4):382. doi: 10.1038/s41419-022-04814-9 (PMC9021253; doi:10.1038/s41419-022-04814-9)
Supplement: Supplementary file 1 — supplement data [file 41419_2022_4814_MOESM1_ESM.pdf]

SFig.1

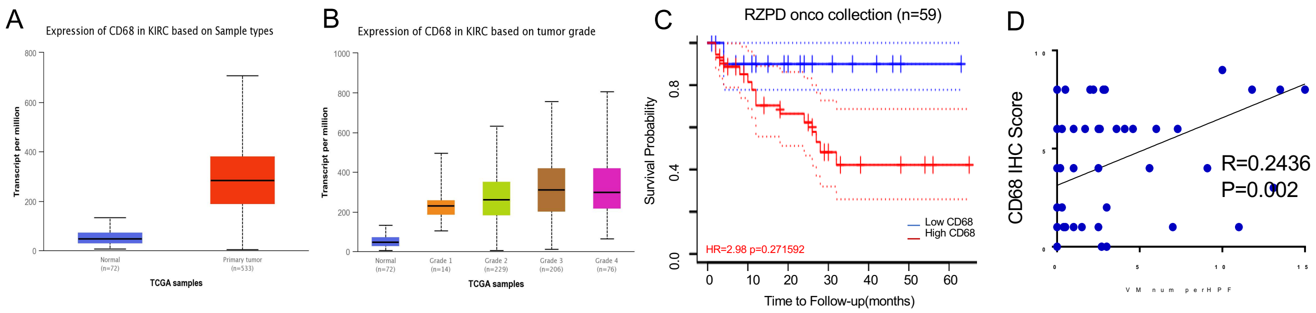

SFig.2

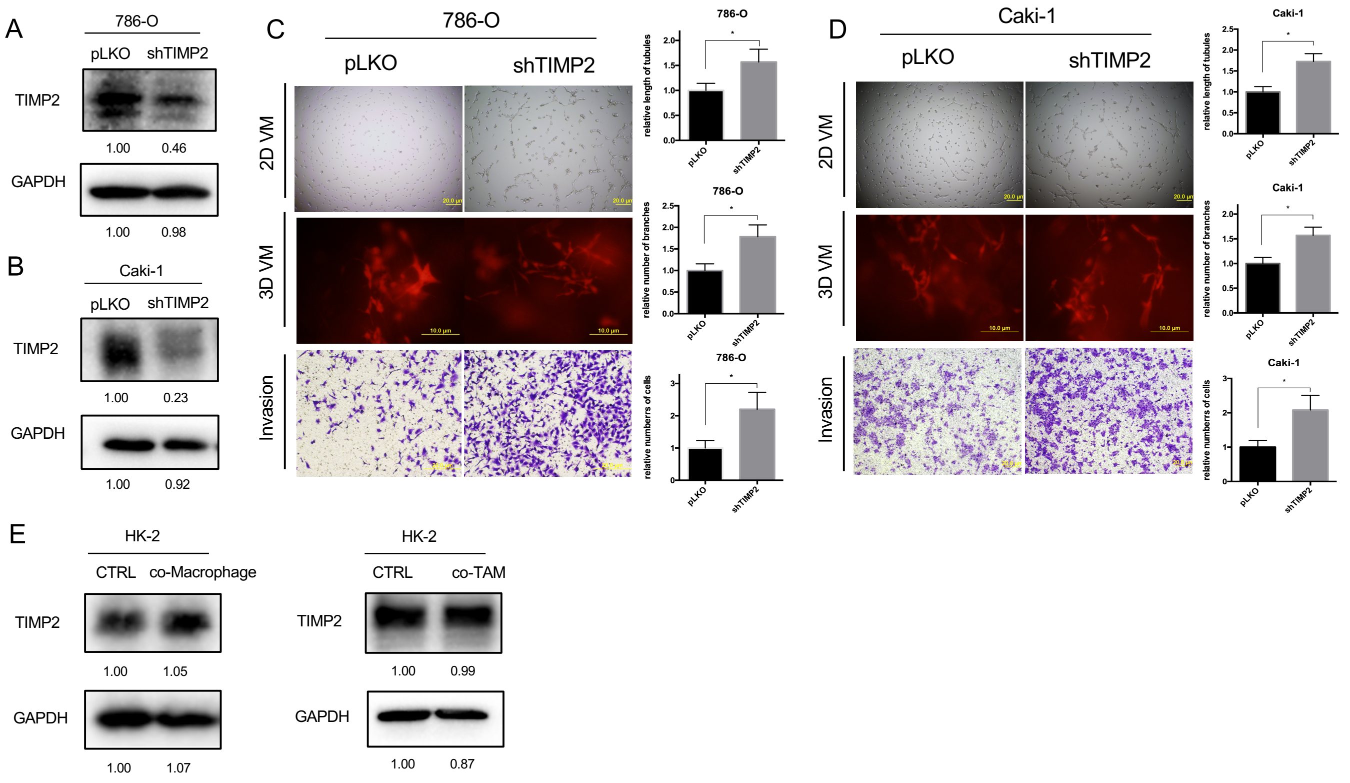

SFig.3

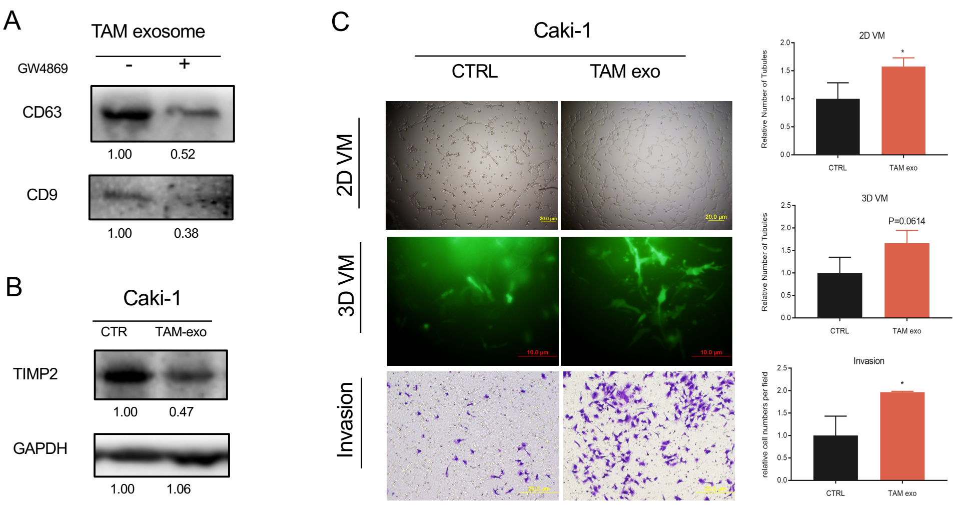

SFig. 1 (A) The comparison of CD68 expression between normal and tumor tissues of RCC patients based on the TCGA database. (B) The expression of CD68 in tissues of different RCC tumor grades based on the TCGA database. (C) The prognostic value of CD68 based on data from The German Cancer Research Center. (D) The correlation between the level of CD68 expression and VM based on analysis of our clinical samples.

SFig. 2 (A-B) Western blot assay was applied to verify the efficacy of TIMP2 knockdown via transfection with pLKO-shTIMP2 in 786-O and Caki-1 cells. (C-D) 2D/3D VM and cell invasion assays were performed in 786-O (C) and Caki-1 cells (D) transfected with shTIMP2 and pLKO control vector. (E) Western blot assay was applied to examine TIMP2 expression in HK-2 cells after co-culturing with macrophages/TAMs.

SFig. 3 (A) Exosome markers CD63 and CD9 were tested by Western blot in TAM-derived exosomes treated with/without exosome inhibitor GW4869. (B) The expression of TIMP2 in Caki-1 cells was tested by Western blot after adding TAM-derived exosomes with PBS as a control. (C) Western blotting was used to detect TIMP2 expression in Caki-1 cells (left) and 786-O cells (right) after adding exosome inhibitor (GW4869) to the coculture

system.

Supplementary Table 1. Patients information

ID	age	sex	location	size cm	lymph metastasis	lung metastasis	grade	TNM	stage
1915551	47	male	right	4.5*3cm	negative	negative	ccRCC	T1bN0M0	1
1898347	42	male	right	6.0*4.8cm	negative	negative	ccRCC	T1bN0M0	1
1847678	49	male	left	6.5*6.0cm	negative	negative	ccRCC	T1bN0M0	1
1635909	68	male	right	7.3*6.0cm	negative	negative	ccRCC	T3aN0M0	3
1249615	50	male	left	6.1*4.8cm	negative	negative	ccRCC	T1bN0M0	1
1633529	38	female	right	6.4*4.8cm	negative	negative	ccRCC	T1bN0M0	1
1251090	53	female	left	5.8*4.4	negative	negative	ccRCC	T1bN0M0	1
1936990	78	female	left	7.7*6.2*6.5cm	negative	negative	ccRCC	T3aN0M0	3
1891533	56	male	left	10*9	negative	negative	ccRCC	T3aN0M0	3
1975161	51	male	left	8.8*11.3cm	negative	negative	ccRCC	T3aN0M0	3
1927048	63	male	left	8.5*7.0cm	negative	negative	ccRCC	T2aN0M0	2
1930642	86	male	right	6*4cm	negative	negative	ccRCC	T3aN0M0	3
1856379	54	male	left	6.5*5.7cm	negative	negative	ccRCC	T3aN0M0	3
1905715	82	male	right	5*4cm	negative	negative	ccRCC	T1bN0M0	1
1675294	52	male	right	5.7*3.9cm	negative	negative	ccRCC	T1bN0M0	1
1215124	59	female	right	8.2*7.7cm	negative	negative	ccRCC	T2aN0M0	2
1228011	49	male	right	3*2.5cm	negative	negative	ccRCC	T1aN0M0	1
1985228	58	female	left	5.3*4cm	negative	negative	ccRCC	T1bN0M0	1
1971417	62	female	left	9.4*8.3*9.4cm	negative	negative	ccRCC	T2aN0M0	2
1927811	39	male	right	6.3*7.7cm	negative	negative	ccRCC	T2aN0M0	2
1938443	54	male	right	9.0*6.1	negative	negative	ccRCC	T3aN0M0	3
1245804	53	female	right	4.6*5.1cm	negative	negative	ccRCC	T1bN0M0	1
1335155	50	female	left	7.44*7.60*6.25cm	negative	negative	ccRCC	T3aN0M0	3
1992193	78	female	right	4.9*4.3cm	negative	negative	ccRCC	T1bN0M0	
1942291	73	female	right	6.7*5.0*5.4cm	negative	negative	ccRCC	T1bN0M0	
2000088	73	female	left	5.5*4.1cm	negative	negative	ccRCC	T1bN0M0	

1937110	67	male	left	5.5*4.5*5.7cm	negative	negative	ccRCC	T3aN0M0	3
	53	female	left	8.5*6.6*6.7cm	negative	negative	ccRCC	T3aN0M0	3
1890759	56	female	left	6*3.5cm	negative	negative	ccRCC	T1bN0M0	
1754627	53	female	left	8.5*6.6*6.7cm	negative	negative	ccRCC	T2aN0M0	2
1890715	47	male	left	5*3.8cm	negative	negative	ccRCC	T3aN0M0	3
1907820	54	male	right	8.6*7.7cm	negative	negative	ccRCC	T2aN0M0	2
1883467	36	male	right	4.6*4.3cm	negative	negative	ccRCC	T1bN0M0	1
2005624	55	male	right	6.0*5.2*5.6cm	negative	negative	ccRCC	T1bN0M0	1
1228555	47	male	right	3.4*4.0cm	negative	negative	ccRCC	T1aN0M0	1
1247108	63	female	left	4.8*3.8cm	negative	negative	ccRCC	T1bN0M0	1
1914167	36	male	left	10.7*10.0cm	negative	negative	ccRCC	T2bN0M0	2
1846487	61	male	right	5.3*4.7*5.2cm	negative	negative	ccRCC	T1bN0M0	1
1230476	65	female	right	9.5*6cm	negative	negative	ccRCC	T2aN0M0	2
1913508	55	male	right	4.8*5.5cm	negative	negative	ccRCC	T1bN0M0	1
1945827	63	male	left	5.0*5.5*5.7cm	negative	negative	ccRCC	T1bN0M0	1
1246857	39	male	left	2.2*2.4cm	negative	negative	ccRCC	T1aN0M0	1
1944047	61	male	right	2.9*3.5cm	negative	negative	ccRCC	T3aN0M1	3
1216468	66	female	right	5.0*5.9cm	negative	negative	ccRCC	T1bN0M0	1
1986144	45	male	left	1.8*2.2cm	negative	negative	ccRCC	T1aN0M0	1
1939470	69	male	right	4.5*3.8cm	negative	negative	ccRCC	T1bN0M0	1
1985448	66	male	left	4.3*4.4cm	negative	negative	ccRCC	T1bN0M0	1
1975770	72	male	left	7*3cm	negative	negative	ccRCC	T1bN0M0	1
2015902	67	male	left	4.5*3.2cm	negative	negative	ccRCC	T1bN0M0	1
1895433	44	male	left	4.2*5.5cm	negative	negative	ccRCC	T1bN0M0	1
1846438	65	male	right	6.9*5.2	negative	negative	ccRCC	T1bN0M0	1

Supplementary Table 2. Antibodies used

ID	Manufacturer	Catalog#
Anti-CD31	Thermo Fisher	MA513188
Anti-CD163	Proteintech	16646-1-AP
Anti-CD68	Proteintech	66231-2-Ig
Anti-TIMP2	Abcam	ab180630
Anti-GAPDH	Santa Cruz	sc-166574
Anti-α-Tubulin	Santa Cruz	sc-423547-LAC
Anti-β-actin	Santa Cruz	sc-517582
Anti-HIF1A	Thermo Fisher	PA1-16601
Anti-VE-cadherin	Santa Cruz	sc-9989
Anti-MMP9	Santa Cruz	sc-393859
Anti-MMP2	Santa Cruz	sc-13595
Anti-Lamc2	Santa Cruz	sc-25341
Anti-EPHA2	ABclonal	A7183
Anti-Nodal	ABclonal	A9902
Anti-Notch4	ABclonal	A8303

Supplementary Table 3. Primers used

ID	Forward primer	Reverse primer
ARG1	5‘-GTGGAA ACTTGCATGGACAAC-3’	5‘-AATCCTGGCACATCGGGAATC-3’
CD163	5’-TTTGTCAACTTGAGTCCCTTCAC-3	5’-TCCCGCTACACTTGTTTTCAC-3’
CD206	5’-GGGTTGCTATCACTCTCTATGC-3’	5’-TTTCTTGTCTGTTGCCGTAGTT -3’
CD68	5’-CTACTTTGCCATCCTTCA-3’	5’-GTGGTTTTGTGGCTCTTGGTA-3’
CD86	5’-CTGCTCATCTATACACGGTTACC-3	5’-GGAAACGTCGTACAGTTCTGTG-3’
NOS2	5’-TTCAGTATCACA ACCTCAGCAAG-3	5’-TGGACCTGCAAGTTA AAATCCC-3’
TNFα	5’-CCTGTAGCCCACGTCGTAGC-3’	5’-AGCAATGACTCCAAAGTAGACC-3’
TIMP2	5’-ACAGGCGTTTTGCAATGCA-3’	5’-GGGTTGCCATAAATGTCGTTTC-3’
HIF1A	5’-AGCTTCTGTTATGAGGCTCACC-3	5’-TGACTTGATGTTCATCGTCCTC-3’
hsa-miR-193a-5p	TGGGTCTTTGCGGGCGAGATGA	
hsa-miR-342-3p	TCTCACACAGAAATCGCACCCGT	
hsa-miR-532-5p	CATGCCTTGAGTGTAGGACCGT	
hsa-miR-423-3p	AGCTCGGTCTGAGGCCCCTCAGT	
hsa-miR-139-5p	TCTACAGTGCACGTGTCTCCAGT	
hsa-miR-423-5p	TGAGGGGCAGAGAGCGAGACTTT	
hsa-miR-125a-3p	ACAGGTGAGGTTCTTGGGAGCC	
hsa-miR-361-3p	TCCCCCAGGTGTGATTCTGATTT	

Supplementary Table 4. Metastasis in mice

mice	liver	intestine	testicle	spleen
NC-1	2		1	
NC-2	3			
NC-3			2	
NC-4				
NC-5	1		1	
NC-6	1(figure 6E)		1(figure 6E)	
inh-1		1(figure 6E)		
inh-2			1	
inh-3				
inh-4				1(figure 6E)
inh-5	1			
inh-6				
