# Supplementary material for: Tumor-associated macrophage-derived exosomes transmitting miR-193a-5p promote the progression of renal cell carcinoma via TIMP2-dependent vasculogenic mimicry
Source: Cell Death Dis. 2022 Apr 20;13(4):382. doi: 10.1038/s41419-022-04814-9 (PMC9021253; doi:10.1038/s41419-022-04814-9)
Supplement: Supplementary file 2 — Original western blot [file 41419_2022_4814_MOESM2_ESM.pptx]

## Slide 1
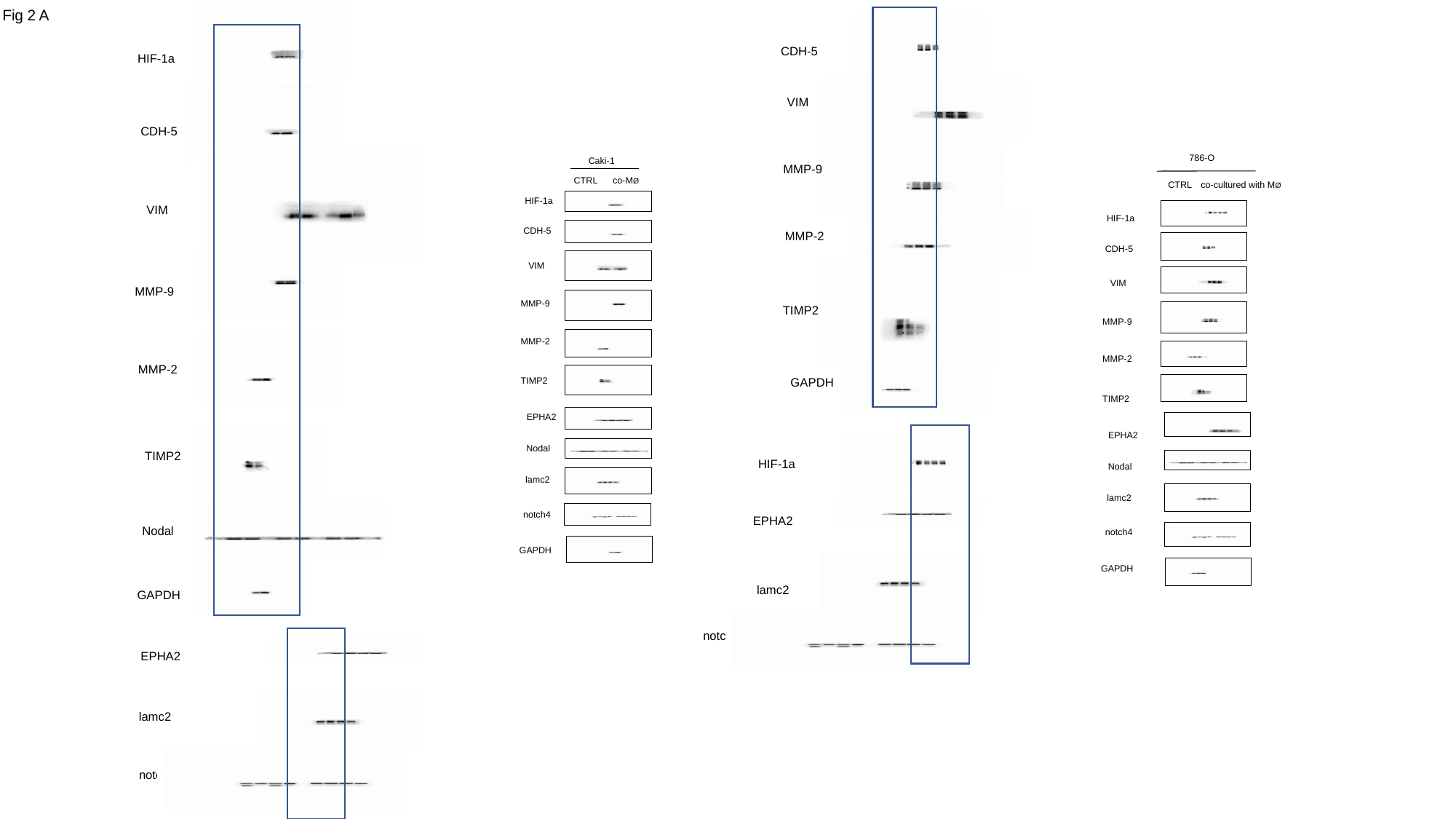

Fig 2 A
HIF-1a
CDH-5
VIM
 CDH-5
786-O
Caki-1
MMP-9
co-MØ
CTRL
CTRL
co-cultured with MØ
HIF-1a
VIM
HIF-1a
 CDH-5
MMP-2
 CDH-5
VIM
VIM
MMP-9
MMP-9
 TIMP2
MMP-9
MMP-2
MMP-2
MMP-2
 GAPDH
 TIMP2
 TIMP2
 EPHA2
 EPHA2
HIF-1a
 Nodal
 TIMP2
 Nodal
lamc2
lamc2
 Nodal
notch4
 EPHA2
notch4
 GAPDH
 GAPDH
lamc2
 GAPDH
notch4
 EPHA2
lamc2
notch4

## Slide 2
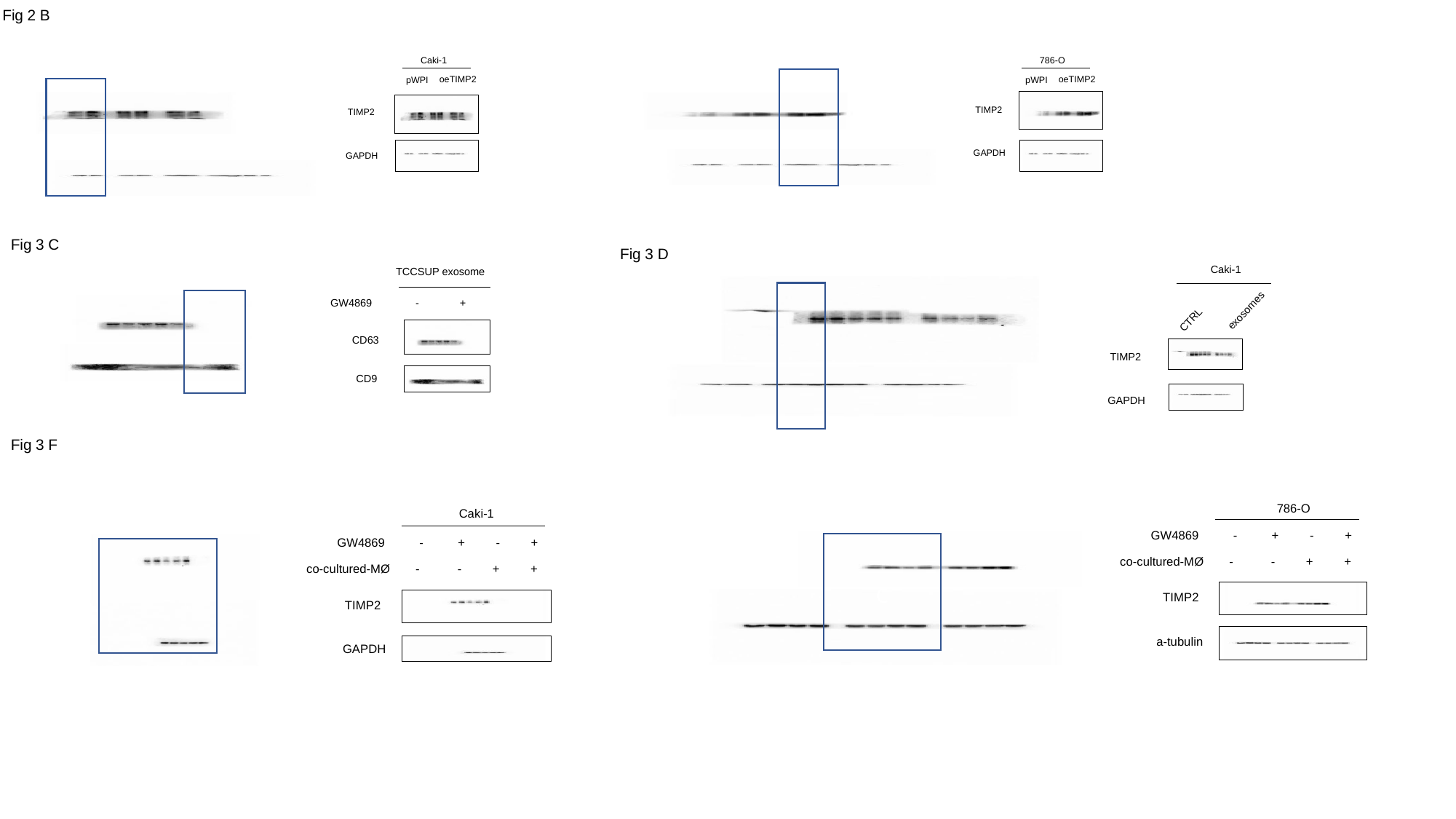

Fig 2 B
Caki-1
786-O
oeTIMP2
oeTIMP2
pWPI
pWPI
 TIMP2
 TIMP2
 GAPDH
 GAPDH
Fig 3 C
Fig 3 D
Caki-1
TCCSUP exosome
GW4869
- +
exosomes
CTRL
 CD63
 TIMP2
 CD9
 GAPDH
Fig 3 F
786-O
Caki-1
GW4869 - + - +
GW4869 - + - +
c
 co-cultured-MØ - - + +
 co-cultured-MØ - - + +
 TIMP2
 TIMP2
 a-tubulin
 GAPDH

## Slide 3
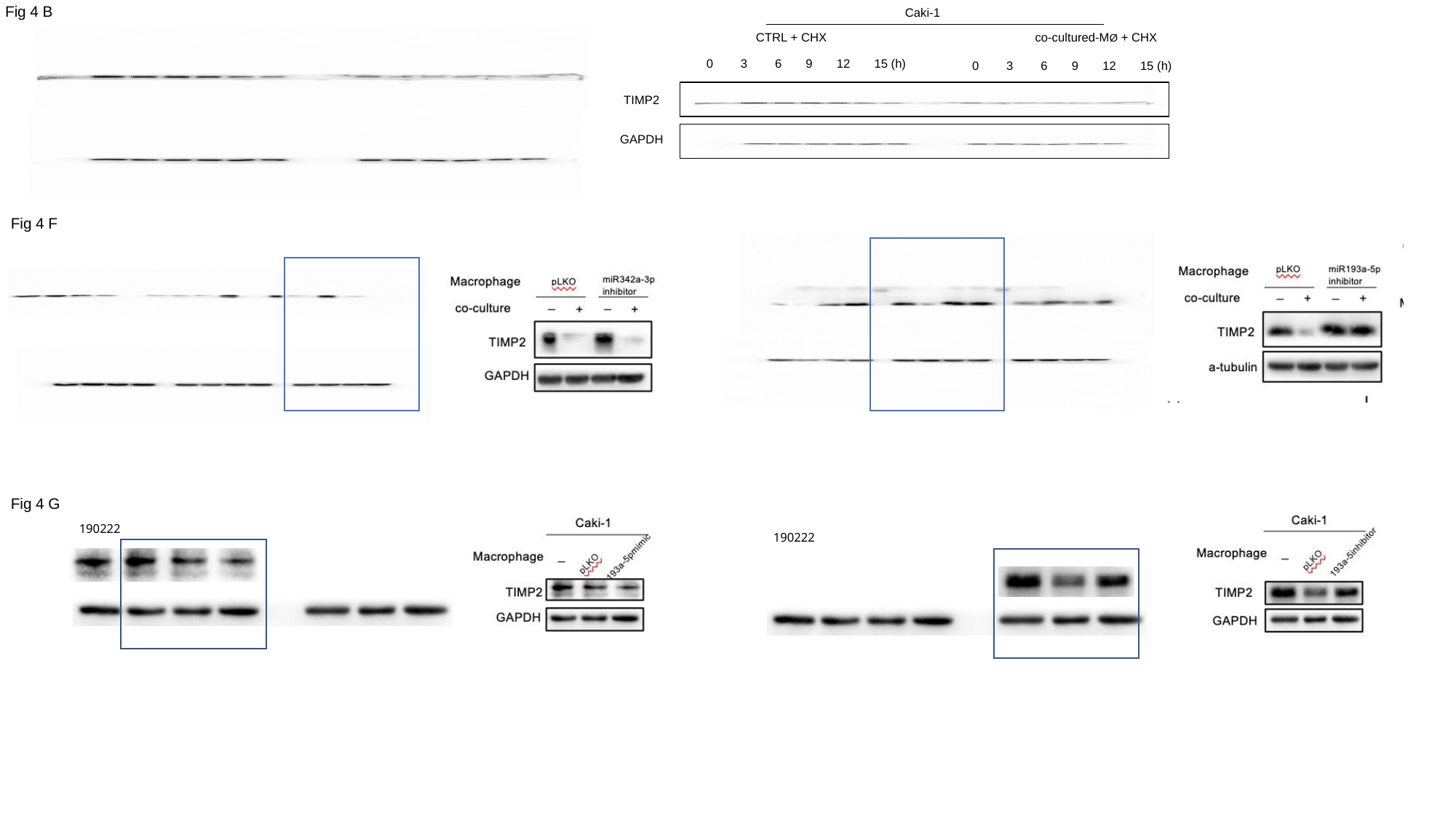

Caki-1
Fig 4 B
CTRL + CHX
co-cultured-MØ + CHX
0 3 6 9 12 15 (h)
0 3 6 9 12 15 (h)
 TIMP2
 GAPDH
Fig 4 F
Fig 4 G
190222
190222
