# Supplementary figures and images for: Correction: Correction to: Tumor-associated macrophage-derived exosomes transmitting miR-193a-5p promote the progression of renal cell carcinoma via TIMP2-dependent vasculogenic mimicry
Source: Cell Death Dis. 2025 Dec 23;16(1):908. doi: 10.1038/s41419-025-08075-0 (PMC12728204; doi:10.1038/s41419-025-08075-0)

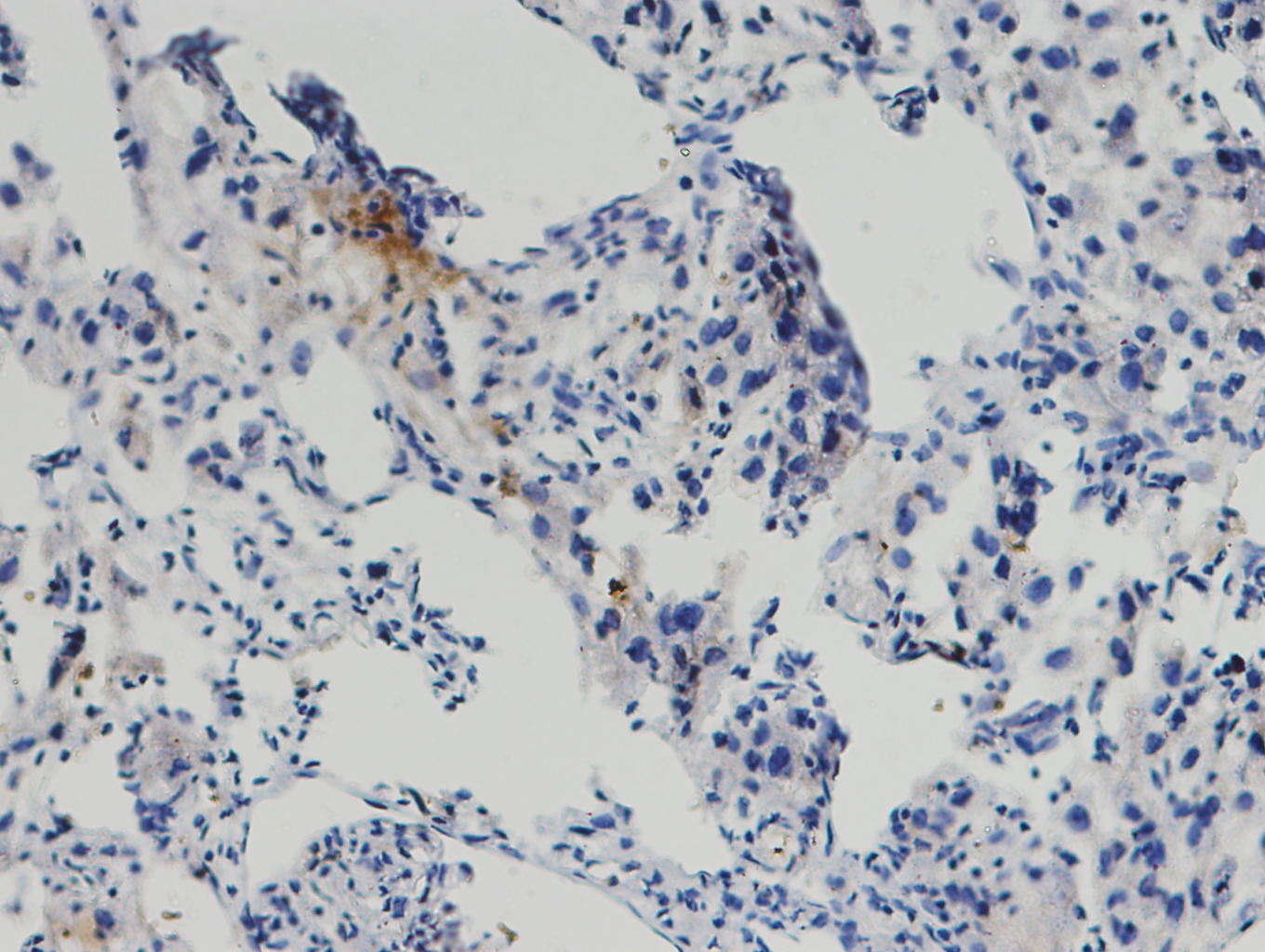

Supplement: Supplementary file 1 — original data of IHC image of CD163 in Fig1A [file 41419_2025_8075_MOESM1_ESM.tif]

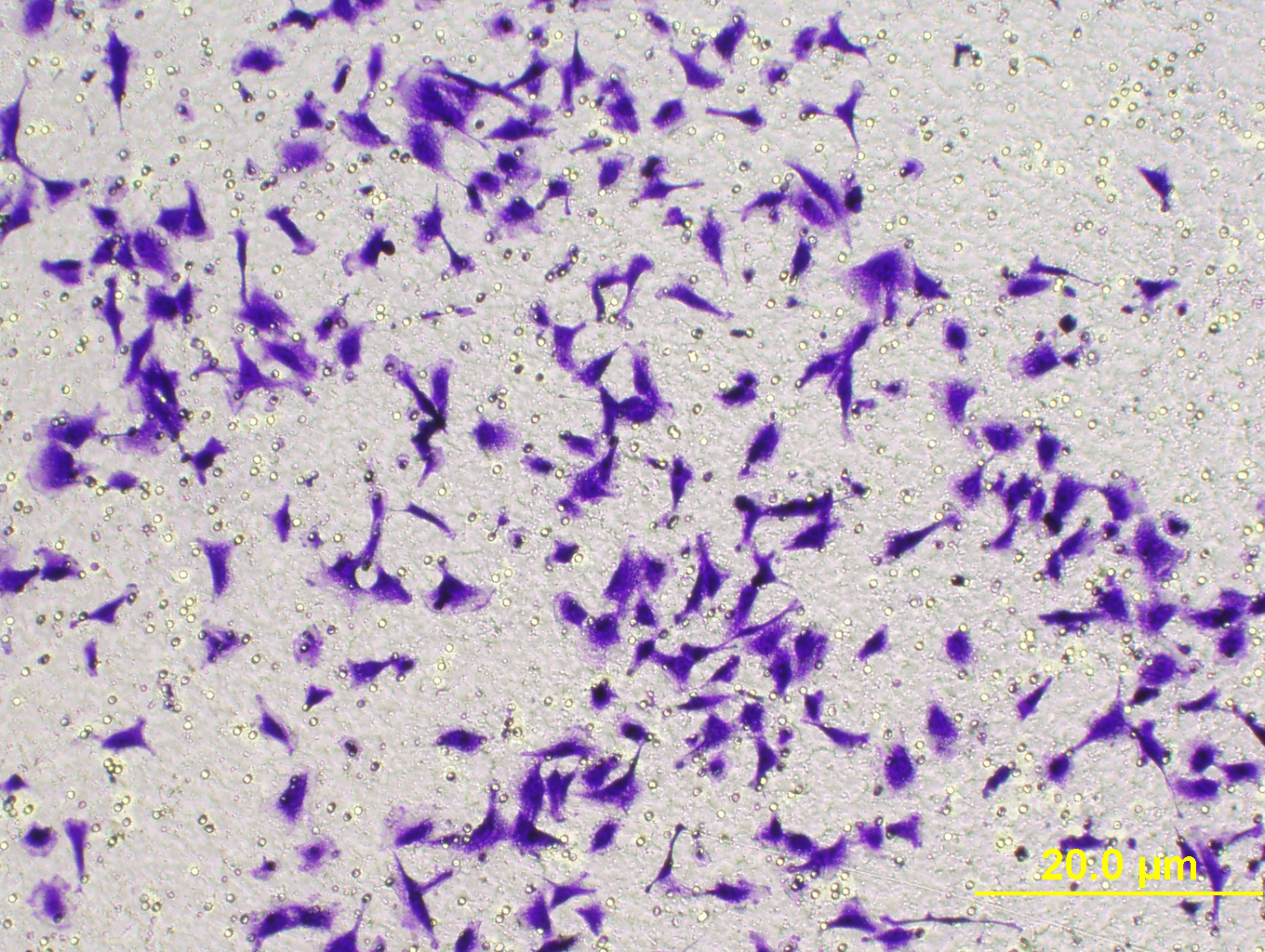

Supplement: Supplementary file 2 — original data of invasion 786-O pwpi coM in fig2D [file 41419_2025_8075_MOESM2_ESM.tif]

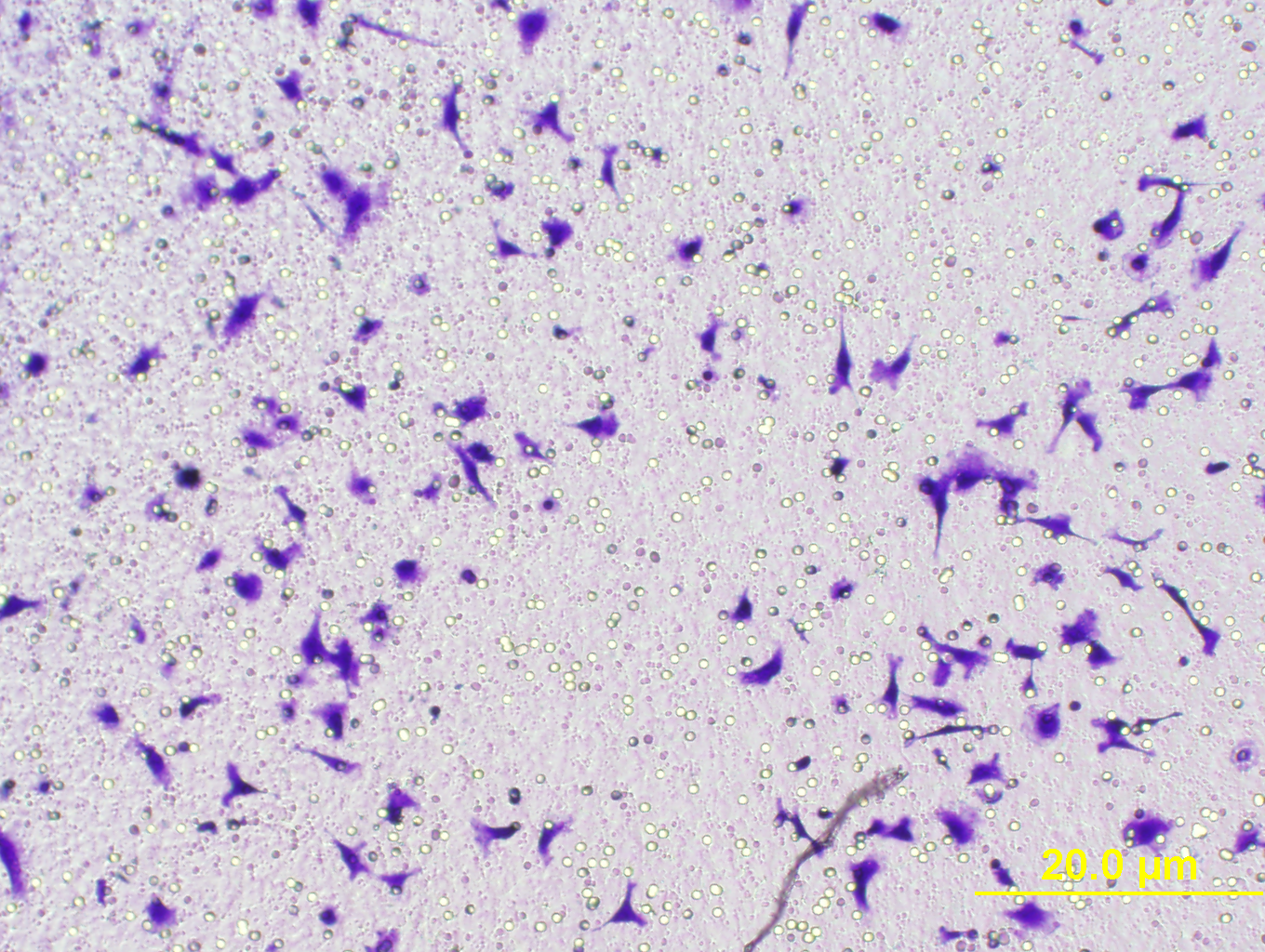

Supplement: Supplementary file 3 — original data of invasion 786-O pwpi in fig2D [file 41419_2025_8075_MOESM3_ESM.tif]
